# HPV Infection and Oxidative Stress in Cervical Carcinogenesis: Linking Apoptosis, Senescence, SASP, and EMT

**DOI:** 10.3390/antiox15040486

**Published:** 2026-04-14

**Authors:** Albert Despot, Rajko Fureš, Ana-Marija Despot, Zlatko Hrgović, Martin Gredičak, Sanja Malinac Malojčić, Vesna Ćosić, Larisa Mešić, Nikola Sinković, Ivan Sabol

**Affiliations:** 1 Clinic for Gynecology and Obstetrics, University Hospital Centre Zagreb, 10000 Zagreb, Croatia; 2 Department of Obstetrics and Gynecology, Zabok General Hospital and Croatian Veterans Hospital, 49210 Zabok, Croatia; rajko.fures@bolnica-zabok.hr (R.F.);; 3 Faculty of Dental Medicine and Health, Josip Juraj Strossmayer University of Osijek, 31000 Osijek, Croatia; 4 Faculty of Health Studies, University of Rijeka, 51000 Rijeka, Croatia; 5 Faculty of Food Technology and Biotechnology, University of Zagreb, 10000 Zagreb, Croatia; 6 Goethe University, 60629 Frankfurt am Main, Germany; 7 Croatian Academy of Medical Sciences, 10000 Zagreb, Croatia; 8 Academy of Medical Sciences in Bosnia and Herzegovina, 71000 Sarajevo, Bosnia and Herzegovina; 9 Polyclinic Ćosić, 35000 Slavonski Brod, Croatia; 10 Department of Gynecology and Obstetrics, General Hospital Tešanj, 74260 Tešanj, Bosnia and Herzegovina; 11 Department of Gynecology and Obstetrics, Faculty of Medicine, University of Zenica, 72000 Zenica, Bosnia and Herzegovina; 12 Croatian Society of Pelviperineology, 49210 Zabok, Croatia; 13 Division of Molecular Medicine, Ruđer Bošković Institute, 10000 Zagreb, Croatia

**Keywords:** ROS, SASP, HPV, cervical cancer

## Abstract

Cervical cancer (CC) is a complex, multistep process involving various viral, molecular, cellular, endogenous, and environmental events that transform normal cervical epithelium into a malignant tumor through a cascade of events. The contribution of high-risk human papillomavirus (HPV) to cancer is significant but involves many additional mechanisms such as oxidative stress (OS), arrested apoptosis of non-functional intraepithelial neoplastic cells, senescence-associated secretory phenotype (SASP), and the final epithelial–mesenchymal transition (EMT) of cervical epithelial neoplasia (CIN) cells. While high-risk HPV oncoproteins E6 and E7 are widely recognized as the primary triggers of CC, the critical role of E6 in degrading the p53 regulatory protein, thereby inhibiting the apoptosis of reactive oxygen species (ROS)-damaged neoplastic cells, is frequently underappreciated in the gynecological literature. Arrested apoptosis of non-functional neoplastic intraepithelial cells is a key event in cervical carcinogenesis and the biological basis of CIN progression via SASP senescence and ultimately EMT. While recent reviews touched upon each of the reviewed aspects, this review aims to provide a general understanding of all links in this complex molecular-biological chain, from HPV infection, oxidative stress, arrested apoptosis, SASP, and EMT. Beyond providing an encompassing primer for clinical researchers, we additionally review potential oxidative stress-related markers for shifting the classic diagnostic and therapeutic paradigms of CIN and cervical cancer.

## 1. Introduction

Cervical cancer remains a significant worldwide health burden and a complex biological phenomenon. Cervical cancer remains among the top most common types of cancer among women [1], with persistent human papillomavirus (HPV) infection identified as the primary risk factor. While HPV is the most prevalent sexually transmitted infection globally [2], the majority of infections are transient and spontaneously cleared by the immune system. However, persistent infections can manifest as premalignant cervical intraepithelial lesions (CIN) which pose a risk for progression to invasive disease [3]. The link between HPV and cervical cancer was established in 1974 by Harald zur Hausen. His team cloned the HPV16 and HPV18 genomes in the mid-1980s and used cervical cancer cell lines to demonstrate the association of the viral genomes E6 and E7 with malignant growth. For the discovery of HPV, Dr zur Hausen received the Nobel Prize in Physiology and Medicine in 2008 [4]. Briefly, the viral oncogene E6 binds to the guardian of the genome, the suppressor protein p53, while the viral oncogene E7 binds to and degrades the retinoblastoma protein (pRb), causing uncontrolled proliferation [5].

While HPV infection is very common, current scientific knowledge indicates that a significant proportion of moderate CIN (CIN2) [6,7] and severe CIN (CIN3) [8] lesions spontaneously regress rather than progress to invasive cancer. Cervical intraepithelial neoplasia is a pathogenic condition closely related to persistent HPV infection, but the virus is not the only driver that contributes to the development of CIN and CC. According to the World Health Organization (WHO) and the International Agency for Research on Cancer (IARC), the incidence of CIN is highest in women between the ages of 20 and 30, especially in low- and middle-income countries [9]. While it is not completely understood why only a fraction of infections ultimately lead to cancer, several of the links have been suggested and are discussed below.

Oxidative stress (OS) is a biological condition characterized by an imbalance between the production of reactive oxygen species (ROS) and the body’s ability to neutralize them with antioxidants. While low levels of ROS are essential for normal cell signaling and immune defense, an excess can damage cellular structures including DNA, proteins, and lipids [10].

The introduction of oxygen into the Earth’s atmosphere has had a dualistic metabolic effect on all eukaryotic organisms since the beginning. Oxygen enabled the evolution of complex organisms with high energy needs, but it is also a constant source of new toxins [11]. The evolutionary shift toward aerobic metabolism provided significant bioenergetic advantages but simultaneously introduced the metabolic burden of reactive oxygen species (ROS). These volatile by-products, most notably the superoxide anion (O_2_^•−^), hydrogen peroxide (H_2_O_2_), and the highly destructive hydroxyl radical (^•^OH), are inherent consequences of mitochondrial oxygen consumption [12]. In recent years, there has been growing interest in researching the joint impact of HPV infection and oxidative stress on the development of cervical cancer [13,14,15,16].

Senescence is a permanent cell cycle arrest. Senescence is usually triggered by DNA damage, a process often referred to as stress-induced premature senescence, and is known to be one of major tumor suppressive effects of p53 [17,18]. Various internal or external stressors can trigger the DNA damage response pathway, thus activating the p53 and/or p16INK4A pathways [19]. The protein p16INK4A plays a key role in the regulation of the cell cycle and aging. It acts as an inhibitor of cyclin-dependent kinases (CDK4, CDK6), blocking phosphorylation of the retinoblastoma tumor suppressor (pRb) and stopping the cell cycle in the G1 phase [20]. Senescence and apoptosis have long been known as directly involved in cervical cancerogenesis [21] and reverting the influence of E6/E7 oncoproteins can also restore the cells toward physiological apoptosis/senescence responses [22,23]. However, senescence remains a biological paradox in cervical carcinogenesis. Through pathways of oncogene-induced arrest, the cell initially blocks the transmission of damaged DNA to subsequent generations. Yet, when senescent cells persist, they undergo extensive metabolic reprogramming and transition into a hyper-secretory state [24]. This chronic senescence-associated secretory phenotype (SASP) induces a pro-tumorigenic niche, characterized by local inflammation and immune exclusion, which collectively fuel the progression from localized CIN to invasive, metastatic disease which has already been implicated in cervical cancer patients survival [25].

The epithelial–mesenchymal transition (EMT) represents a profound cellular reconfiguration, wherein polarized epithelial cells, anchored to the basement membrane, shed their architectural constraints. Through a series of coordinated biochemical shifts, these cells adopt a mesenchymal state characterized by a transitioned phenotype with elevated apoptosis resistance, migratory capability, and invasiveness [26].

EMT involves the alteration of cell types and several transcription factors have been implicated in the regulation of gene expression related to this transition. Studies of transcriptional regulators in the evolution of precancerous lesions of the cervical epithelium are not very common, but fortunately they exist. One such study identified the transcription protein SNAIL as an important transcription factor that regulates cervical cancer invasion [27]. Another recent study revealed a gradual increase in the transcriptional markers TWIST, SNAIL, and SLUG as precancerous lesions progressed from LSIL to HSIL [28].

The topic of this article is not only a description of the transformation of CIN into CC, because without mentioning all the actors who contributed to it, this overview would not be complete. Cervical cancer, due to its anatomical location, visualization capabilities, direct access, and significant impact on women’s health, has attracted the attention of experts and scientific researchers since the late 19th century. This review summarizes the impacts of HPV infection, oxidative stress, reactive oxygen species, senescence-associated secretory phenotype (SASP), and epithelial–mesenchymal transition (EMT), likely the most important links in the complex molecular-biological chain known as cervical cancer.

Undeniably, each of those topics has been reviewed previously in the context of HPV [13,15,29,30,31,32,33,34,35]; however, this review integrates all critical steps of HPV-driven cervical carcinogenesis into a comprehensive and coherent overview, with oxidative stress as a central unifying theme, intended as a structured starting point for researchers entering the field.

## 2. HPV as a Major Factor in the Development of Cervical Cancer

HPV is the initiator of complex molecular-biological events that transform normal cervical epithelium into CIN. HPV is undoubtedly the trigger for all these events, but without the help of ROS and chronic infection, cervical cancer as the ultimate pathological condition is difficult to achieve in vivo. Unfortunately, there are numerous conditions from a woman’s lifestyle that contribute to the development of CC, and as a rule, pass under the radar of all screening programs [13]. Human papilloma virus itself (HPV) is a small, non-enveloped, double-stranded DNA virus with a tropism for squamous epithelium. It is a member of the Papilloma viridae (PV) virus family, which, as of 2019, is divided into 53 genera, each denoted by a letter of the Greek alphabet [36]. Only five genera of papilloma viruses can infect humans. Currently there are 231 recognized HPV types (updated information can be found at the Papillomavirus Episteme [37]) and they are often classified into low-risk (LR-HPV, i.e., 6 and 11, etc.) and high risk (HR-HPV, i.e., 16, 18, 45, etc.) types depending on their association with cervical cancer [38].

HPV infection is characterized by a strict requirement for the metabolically active, undifferentiated environment found at the base of the squamous epithelium [39]. Local tissue architecture, comprising a gradient from basal progenitors to cornified squamous cells, presents a challenge for a virus that does not encode for its own polymerases [40]. To facilitate its genome replication, HPV must prioritize the infection of the basal layer, thereby bypassing the replicative ‘dead end’ of differentiated suprabasal cells that have transitioned out of the active cell cycle [41]. Access to the proliferative basal compartment is typically facilitated by physical micro-trauma or mucosal abrasions [42]. However, the cervical transformation zone (TZ) presents a unique physiological bypass to this requirement. In this transitional landscape, where the simple columnar cells of the endocervix meet the stratified squamous architecture of the ectocervix, the basal layer is naturally more accessible to viral entry without the necessity of mechanical injury. Furthermore, this junctional niche harbors specialized ‘reserve cells’, a population of resident stem cells that serve as primary targets for high-risk HPV, potentially acting as the cellular origin for persistent oncogenic transformation [43]. Accounting for a significant portion of the global cancer burden, high-risk HPV types (such as 16, 18, and 31) leverage their early genes to disrupt host cellular homeostasis [44]. The transformation potential of these viruses lies in the early-region proteins, specifically E6 and E7, which induce uncontrolled mitotic entry. In the cervical niche, this viral pressure collides with oxidative stress, creating a lethal combination that promotes protein carbonylation and DNA damage. This process is further intensified by the E6-mediated sequestration of p53, which prevents the apoptosis of damaged cells [45,46,47,48]. Beyond genomic disruption, HPV oncoproteins are known to influence cell metabolism [49], shifting the glucose fermentation to lactate which can lead to electron transport chain inefficiencies in mitochondria and subsequent ROS accumulation [50]. The persistence of these high-risk types also facilitates a shift in the local cytokine profile; the upregulated production of TGF-β1 and IL-10 creates a sheltered, immunosuppressive environment that permits the progression from dysplasia to invasive cancer. While pathogenesis is primarily driven by E6 and E7, the often-overlooked E5 oncoprotein also plays a critical synergistic role [51]. E5 facilitates early cellular transformation by hyperactivating EGFR signaling but also has direct roles in the inhibition of apoptosis as well as immune evasion [52,53,54]. Cervical cancer progression is also known to be associated with chronic inflammation [55]. The inflammatory landscape of high-risk HPV infection is largely dictated by the viral oncoproteins (E5, E6, and E7), which aggressively stimulate the cyclooxygenase-2 (COX-2) and prostaglandin E2 (PGE2) cascade. This specific molecular axis is widely recognized as the central instigator of the viral inflammatory response. Beyond the COX-PGE2 axis, the continuous expression of these viral genes directly triggers the release of a diverse array of pro-inflammatory cytokines [56]. Ultimately, this cytokine amplification creates a hostile, inflamed microenvironment that accelerates the transition from a simple viral infection to established dysplasia [55].

**Figure 1:**
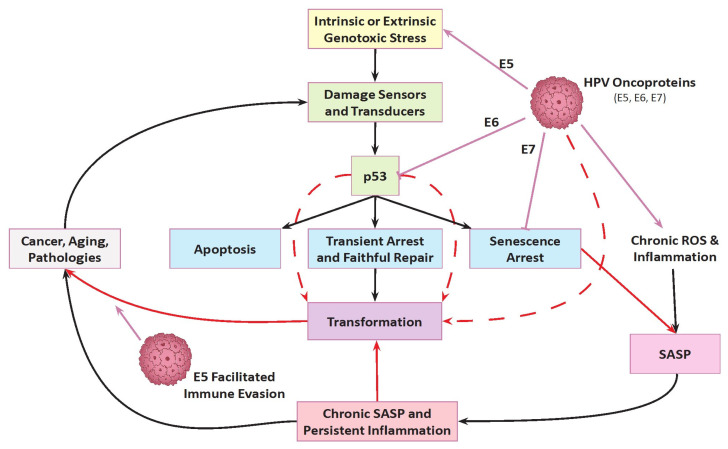
The synergistic mechanistic pathway of HPV-driven cervical carcinogenesis. Following high-risk Human Papillomavirus (HPV) infection, the viral oncoproteins E5, E6, and E7 cooperatively dismantle cellular homeostasis. E6 and E7 drive canonical oncogenic pathways via the targeted inhibition of tumor suppressors p53 and pRb, resulting in impaired apoptosis and cell cycle deregulation. Concurrently, the E5 oncoprotein acts as a critical amplifier by hyperactivating Epidermal Growth Factor Receptor (EGFR) signaling and upregulating the COX-2 inflammatory axis. These combined viral disruptions converge to generate high levels of Reactive Oxygen Species (ROS). The resulting chronic oxidative stress and uncorrected DNA damage trigger cellular senescence in the surrounding tissue leading to the accumulation of cells exhibiting a Senescence-Associated Secretory Phenotype (SASP). SASP paracrine signaling and persistent inflammation alters the microenvironment and allows cells with robust E6/E7 expression, which can actively bypass this senescent arrest as well as apoptosis (dashed red lines), to undergo further dysplasia progression. Ultimately, epithelial–mesenchymal transition, complemented by E5-mediated immune avoidance, culminate in invasive cervical cancer.

HPV infection, Reactive Oxygen Species (ROS), and chronic inflammation form a feed-forward loop that drives cervical cancer, where persistent HPV triggers inflammation and ROS, leading to cellular damage, DNA mutations, and malignant transformation, a process accelerated by immune response failure and sustained inflammatory signaling [57,58] (Figure 1). Despite clinical advancements in prophylactic immunization, surgical interventions, and chemotherapeutic regimens, the holistic management of cervical malignancies continues to face critical limitations. Most notably, while current HPV vaccines offer robust primary prevention against incident viral exposure, they are strictly prophylactic; they impart no therapeutic benefit, viral clearance, or disease regression for individuals harboring established infections or pre-existing neoplastic lesions [59]. Among the challenges that researchers will face in the coming time is the need to explain the origin of subclinical HPV infections [60] as the age-specific incidence of cervical cancer currently shows a bipolar pattern in some high-income countries like Denmark [61] and USA [62]. In most cases of unclear subclinical or chronic latent HPV infections, or sudden onset of cervical cancer in postmenopause, the cause of development might be found in a viral infection of basal epithelial cells, unrecognized by the PAP test, which is arrested in a state of senescence (stable cell cycle arrest) and reactivated after 15–20–30 years.

Cervical intraepithelial neoplasia (CIN) is a summary term for abnormal growth of squamous cells of the cervix that historically encompassed mild (CIN1), moderate (CIN2), or severe (CIN3) lesions. It is routinely identified by pathohistological evaluation by biopsy [63]. As mentioned above, HPV is necessary for the development of CIN starting with mild lesions which produce new virions [64] but is only one of the factors required for the disease progression towards cervical cancer. In most cases, the lesions progress rarely as well as slowly taking even decades from infection to cancer [65]. This long period makes combating invasive cancer through screening effective and organized screening programs made significant reductions in mortality possible even with less sensitive methods [66]. Despite more than 100 years of research into cervical neoplasia, the issue of identifying causes of progression to cancer remain unresolved as the major efforts were directed towards HPV and possibly other aspects remain unexplored to date.

## 3. Oxidative Stress (OS) and Reactive Oxygen Species (ROS)

Oxidative stress (OS) is an imbalance where the body produces too many unstable molecules called free radicals (reactive oxygen species or ROS) and/or lacks enough antioxidants to neutralize them, leading to cellular and tissue damage [10]. Mechanistically, chronic infections are well-established inducers of oxidative stress (OS) via inflammatory pathways. Consistent with this paradigm, persistent HPV infection acts synergistically with OS, generating high levels of endogenous and exogenous ROS, such as superoxide anion (O_2_^•−^) and hydrogen peroxide (H_2_O_2_) [13,67]. This suggests that OS itself may be the chronic trigger facilitating the progression of HPV from a simple viral infection to LSIL, HSIL, and ultimately invasive cancer.

Reactive oxygen species (ROS) exhibit a dual physiological nature; though continuously produced via basal metabolic pathways, they are also essential mediators of homeostatic cellular signaling [12]. Cellular fate in the presence of ROS is dictated by the ratio between the oxidative burden and endogenous antioxidant reserves. When this homeostasis is disrupted, the intracellular environment shifts into oxidative stress (OS), culminating in damage to the membrane lipids, cellular proteins, and nucleic acids [68].

The number of sites within each cell where superoxide anion (O_2_^•−^) and H_2_O_2_ are generated is significant, regardless of whether this production is physiological or abnormal. Superoxide anion (O_2_^•−^) and H_2_O_2_ are the most important ROS members, co-creators of cellular homeostasis, but also generators of numerous abnormal conditions, including cancer. The analysis of essential ROS molecules at the structural and functional level is the basis for understanding all causal events within oxidative stress and has been a focus of recent research. ROS are molecules with unpaired electrons or labile O-O bonds (superoxide anion, hydrogen peroxide, hydroxyl radical, singlet oxygen) which react easily with other molecules. Both O_2_^•−^ and H_2_O_2_, may, however, play a dual role. At the level of physiological balance, ROS intermediate molecules (O_2_^•−^, H_2_O_2_) formed in the process of reducing molecular oxygen to water are very important for cellular signaling and homeostasis [69], and at the pathological level they are considered harmful because they can facilitate the generation of highly reactive species such as hydroxyl radicals (^•^OH) which lead to damage causing abnormal conditions such as neurodegenerative diseases, atherosclerosis, hypertension, cardiovascular diseases, diabetes mellitus, vitiligo, bipolar disorder, schizophrenia, cancer, and degenerative diseases associated with aging [70].

The relationship between oxidative stress, excess reactive oxygen species, and HPV infection is quite complex [15] and further research is needed to fully understand its nuances. Recent research suggests that ROS (superoxide anion, hydroxyl radical) and HPV infection may have the same targets inside the cell, acting together or independently: (i) tumor suppressor protein p53, (ii) TP53 gene, (iii) tumor suppressor protein pRb, (iv) mitochondrial membrane complexes I, III, and IV, (v) NADPH oxidase (NOX 1–5; DUOX 1–2), (vi) xanthine oxidase (XO), (vii) cyclooxygenase 2 (COX-2), (viii) ATM (ataxia and telangiectasia mutated) kinase, (ix) ATR (ATM- and Rad3-related) kinase, (x) DNA-PK (DNA-dependent protein kinase), and (xi) PARP-1 (poly ADP-ribose polymerase 1) [71,72,73]. Furthermore, the membranes of cellular organelles, including the endoplasmic reticulum (ER), lysosomes, and peroxisomes, are highly vulnerable to these combined effects.

One of the primary challenges in fully understanding the role of ROS is the lack of effective methods for their in vivo detection, mainly due to their very short lifespan and the presence of enzymatic antioxidant systems in cells. ROS have short half-lives (t_1/2_: milliseconds to nanoseconds), and their migration distances or radius of action are measured in micro or nanometers [74] (Table 1). Despite these detection challenges, it is clear that endogenous ROS, generated by HPV alone or amplified by chronic inflammation, closes the circle of causal events that ultimately induce the transition of intraepithelial neoplastic cells into invasive cancer.

### 3.1. Superoxide Anion (O_2_^•−^)

Superoxide anion (O_2_^•−^) is the largest generator of H_2_O_2_ in the metabolism of every eukaryotic cell. In mammals, it is very important because, in addition to being a regulator of homeostasis, it participates indirectly in the development of many diseases, including cancer, via H_2_O_2_ and Fenton’s reaction. Superoxide anion is a reduced form of molecular oxygen with a negative electrical charge which acts as both a radical and an anion [75].

Unpaired electrons make free radicals as well as superoxide anion highly reactive and able to oxidize organic molecules [76,77].

In biological contexts, O_2_^•−^ is produced as a byproduct in the electron transport chain (ETC) during mitochondrial (cellular) respiration. Approximately 2% of the oxygen consumed is partially reduced to superoxide anion rather than fully to water [71]. In the mitochondrial electron transport chain O_2_^•−^ is the initial product of incomplete oxygen reduction, acting as both a signaling molecule, and if accumulated a potential toxin. Both O_2_^•−^ and H_2_O_2_ are in fact selective in their reactions with biological molecules allowing their signaling functions, while [^•^OH] indiscriminately damages DNA and proteins. To prevent cellular damage, enzyme-like superoxide dismutase (SOD) rapidly convert superoxide anion into hydrogen peroxide which is then further reduced to water by catalase (CAT) and glutathione peroxidase (GPx) [71,78].

The problems rise when the production of ROS due to aging or lifestyle factors exceeds the action of enzymatic defenses leading to oxidative stress [10]. Metabolically, O_2_^•−^ is intimately tied to mitochondrial respiration and specific cellular enzymes. Due to the importance of the superoxide anion in overall cellular metabolism, it is critical to highlight its primary mitochondrial and enzymatic sources:

(i) Cytochrome c oxidase (Complex IV): While a key component of the respiratory chain [15,79,80], Complex IV is a large and complex multi-protein assembly, primarily acting to safely bind and fully reduce O_2_ to water. Because partially reduced oxygen species are damaging, its high affinity for oxygen—allowing it to operate at tissue O_2_ levels as low as 5 to 15 mmHg—functions fundamentally as an antioxidant defense to minimize ROS leakage. The four redox-active metal centers (Cu_A_, Cu_b_, haem a, haem a_3_) in cytochrome c oxidase play key roles in both O_2_ reduction and safe binding.

(ii) NADH:ubiquinone oxidoreductase (Mitochondrial Complex I): The largest enzyme in the electron transport chain within mitochondria acting as the primary entry point for electrons from NADH, Mitochondrial complex I acts as a major source of ROS. Defects of Complex I are linked to numerous diseases, including mitochondrial disorders [81]. While the primary function of mitochondria is the synthesis of adenosine triphosphate (ATP), this bioenergetic process is inherently imperfect. During oxidative phosphorylation, electron ‘slippage’ along the respiratory chain and its accessory enzymes can result in superoxide O_2_^•−^ or H_2_O_2_ [82]. Electrons can also leak from the flavin mononucleotide (FMN) site or iron-sulfur clusters via highly reduced flavin radical intermediate [83]. Mammalian mitochondria failures can occur at several sites in the electron transport chain that lead to O_2_^•−^ and/or H_2_O_2_, both within or outside of the inner mitochondria membrane [84,85]. It warrants repeating that HPV infection itself can adversely affect mitochondria morphology and further increase ROS production above natural levels [50].

(iii) Cytochrome bc_1_ complex (Mitochondrial Complex III): Complex III receives high-energy electrons from ubiquinol. During the Q-cycle, it acts as the second major source of mitochondrial ROS, specifically releasing of O_2_^•−^ at the Q_o_ site, which functions as a signaling molecule in processes like oxygen sensing and immune regulation [86,87].

(iv) NADPH oxidase (NOX): Unlike mitochondria, where O_2_^•−^ and H_2_O_2_ are respiratory by-products, the NOX family of transmembrane enzymes (including NOX1–5 and DUOX1–2) exists primarily to produce ROS for immunity and cell signaling [88]. Deregulation of these enzymes, and the subsequent failure to control O_2_^•−^ and H_2_O_2_ activity, is a recognized trigger for numerous pathological disorders, including cancer and chronic inflammation [89].

(v) Xanthine oxidase (XO): XO is a metalloenzyme containing molybdenum, iron, and the cofactor FAD [90]. Uniquely possessed by mammals for the catabolism of hypoxanthine and xanthine into uric acid [91,92], XO acts as a potent generator of ROS and likely has more than a purely theoretical contribution to the development of cervical cancer.

Interestingly, HPV infection was found to lead to metabolomic shifts with direct influence on the above sources of ROS [50] and directly modulate ROS production [93,94]. Of note is the direct interaction of high-risk HPV18 E2 early protein with mitochondrial membranes and components of the respiratory chain [95]

### 3.2. Hydrogen Peroxide (H_2_O_2_): A Key Metabolic Molecule with a Significant Impact on Quality of Life and Evolutionary Development

Oxygen (O_2_), superoxide anion (O_2_^•−^), hydrogen peroxide (H_2_O_2_), and water (H_2_O) are the basic molecules on which the entire metabolic architecture of the biological world is built, not only structurally and functionally, but also developmentally. The acquisition of electrons by O_2_ results in the formation of O_2_^•−^, which will further generate other ROS [96] unless the enzyme superoxide dismutase (SOD) can return it to its original state by taking away an electron. SOD is a crucial antioxidant enzyme that protects cells by catalyzing the self-reaction of superoxide radical (O_2_^•−^) converting it into less harmful molecular oxygen (O_2_) and hydrogen peroxide (H_2_O_2_) [97].

In the cellular metabolism of all aerobic organisms, hydrogen peroxide is a very important molecule, but its dualistic character is relatively unknown, especially in the world of clinical medicine. Hydrogen peroxide is a molecule that, at low concentrations and in the environment of an efficient enzymatic antioxidant system, is the main driver of a complex system of intracellular signaling and homeostasis in all spheres of life [71,98,99,100,101]. Primarily because of its specific physicochemical properties, namely a longer half-life and greater migration distance compared to other ROS (Table 1), H_2_O_2_ is uniquely capable of serving as an intracellular messenger carrying redox signals from the site of its generation to distant sites [102]. Under physiological conditions (eustress), H_2_O_2_ is most suitable for redox signaling via the control of enzymatic activity and is known to modulate broad regulatory network, directly influencing critical transcription factors such as NF-κB, HIF-1, TP53, NRF2, CREB, and AP-1 [98]. The fundamental nature of this redox regulatory paradigm is underscored by its evolutionary conservation with similarity in simple eukaryotes (Yap1, Hsf1, Maf1, and Msn2/4) and bacteria (PerR and OxyR) [98,103]. Interestingly, HR-HPV infections have been shown to affect the H_2_O_2_ levels in the vaginal microenvironment, albeit likely by increasing the abundance of H_2_O_2_ producing bacteria [104].

However, when cellular antioxidant defenses fail, the dualistic character of this molecule shifts toward distress. In these conditions, excess H_2_O_2_ acts as a constant generator of oxidative damage and drives numerous pathological conditions, including cancer [98,105,106,107]. Given the importance of H_2_O_2_ in the metabolism of every eukaryotic cell, its specific chemical structure and genesis warrant closer examination.

#### 3.2.1. The Chemical Structure of H_2_O_2_

Hydrogen peroxide (H_2_O_2_) is a small molecule similar to water. Its size allows it to diffuse easily through specific membrane channels called peroxyporins (a subgroup of aquaporins). Contrary to traditional assumptions of passive lipid diffusion, the movement of extracellular into the cellular interior is heavily dependent on aquaporin channels, compensating for the molecule’s weak membrane permeability [108,109,110]. Interestingly, the channels themselves are modulated by changes in the redox environment [111]. Elucidating the specific role of these transport proteins represents a necessary step forward in the study of oxidative stress pathways [112].

While strictly classified as a non-radical molecule, H_2_O_2_ possesses an intrinsic structural instability that makes it highly reactive within a biological context. Unlike the superoxide anion, it lacks an unpaired electron; however, its inherent polarity and weak internal bonds prime it for rapid metabolic interactions. Most critically for cellular pathogenesis, H_2_O_2_ reacts with intracellular Fe^2+^ at sub nanosecond speeds, asymmetrically decomposing into hydroxyl radical [^•^OH] and hydroxide anions [OH]^−^.

#### 3.2.2. Hydrogen Peroxide and the Fenton Reaction

The redox regulation of H_2_O_2_ and its relationship with the iron ion (Fe^2+^) is a complex process. It is fascinating that the simple reaction between Fe^2+^ and H_2_O_2,_ first observed by H.J.H. Fenton over 125 years ago, remains so complex to fully characterize in vivo [113,114], despite H_2_O_2_ being a key molecule of intracellular signaling and antioxidants generation [115,116]. Crucially, H_2_O_2_ in high concentrations can quickly become a major driver of pathological ROS via the Fenton reaction, a mechanism often underappreciated in clinical oncology [117,118,119]. The classic Fenton reaction is defined as:

(1) Fe^2+^ + H_2_O_2_ → Fe^3+^ + [OH]^−^ + [^•^OH]

The mechanism of the Fenton reaction has been studied and well summarized by Stanbury [120]. The Fenton reaction is understood to be highly dependent on environmental pH and iron concentration, producing oxoiron (IV) species at pH > 3 and the highly destructive [^•^OH] under more acidic conditions [121,122,123].

While the chemical formula for the Fenton reaction is identical in inorganic and biological environments, the conditions of occurrence, auxiliary factors, control mechanisms, and consequences of the same reaction in the living and non-living world are diametrically opposed [124]. In human cell metabolism, the toxic potential of the Fenton reaction is heavily context-dependent. When Fe^2+^ is safely bound within the heme group of a hemoglobin molecule or a cytochrome enzyme, the Fenton reaction is heavily restricted. However, as free, unbound iron accumulates in the tumor microenvironment, its toxic potential is unleashed [118,125]. On the other hand, women with high levels of ferritin were less likely to clear incident HPV infections [126] but the association of iron intake and HPV infections is non linear [127].

In evolutionary development over billions of years, nature did not accidentally choose iron (Fe) as the main factor in redox processes. Because free transition metals are highly reactive, cellular biochemistry evolved to safely regulate their catalytic activity by incorporating them into cyclic porphyrin rings. The quintessential human example is heme, a metalloporphyrin formed when protoporphyrin IX coordinates a central Fe^2+^ ion [128]. This structural motif acts as the functional core for a diverse network of critical cellular proteins. Beyond its structural role in oxygen-binding proteins (hemoglobin, myoglobin), heme provides the catalytic engine for major redox-active enzymes, encompassing the cytochrome p450 monooxygenases, catalase, peroxidases, and various cytochromes.

## 4. Enzymatic Antioxidant Defense System

While physiological levels of the superoxide anion (O_2_^•−^) and hydrogen peroxide (H_2_O_2_) participate in redox signaling, their production is significantly increased during conditions of oxidative stress (OS), potentially disrupting the homeostasis [105,129]. To prevent macromolecular damage, cells rely on a highly coordinated enzymatic antioxidant defense system and the review will focus on the enzymes possibly involved in CIN and cervical cancer [130,131]. The primary, first-line enzymes include Superoxide Dismutase (SOD) for neutralizing O_2_^•−^, alongside Catalase (CAT), Glutathione peroxidase (GPx), and Peroxiredoxins (PRDX) for breaking down hydrogen peroxide (H_2_O_2_) [131,132,133].

Superoxide Dismutase (SOD) family comprises metal-containing enzymes that act as the first line of defense by converting O_2_^•−^ into oxygen and H_2_O_2_ through a disproportionation reaction [134]. To exert maximum catalytic activity, SOD requires metal cofactors (iron, manganese, or copper/zinc) that donate electrons and regenerate throughout the catalytic cycle [134]. In eukaryotes, SODs are classified by their cofactors and cellular localization: copper–zinc SOD (Cu/Zn-SOD or SOD1) in the cytoplasm, manganese SOD (Mn-SOD or SOD2) in the mitochondrial matrix, and extracellular SOD (EC-SOD or SOD3) [135,136]. Together, they ensure that highly toxic superoxide radicals generated by localized metabolic leaks or NOX activity are rapidly converted into H_2_O_2_ for subsequent neutralization [137,138].

Catalase (CAT) acts as a cornerstone of the aerobic antioxidant defense system, rapidly clearing reactive intermediates in organs with high metabolic demands. Its enzymatic proficiency not only prevents localized OS damage but also regulates broader biological processes, including immune signaling and cellular aging [131,139,140]. Given its central role in maintaining systemic redox equilibrium, CAT dysfunction is deleterious [141]. Structurally, CAT is a complex tetrameric protein containing four heme groups with iron ions that enable rapid reaction with peroxides [142]. Catalase breaks down two H_2_O_2_ molecules into water and oxygen [143,144]. To manage H_2_O_2_ homeostasis, and prevent it from fueling the toxic Fenton reaction, CAT exhibits an extraordinary turnover rate, converting millions of H_2_O_2_ molecules per second [145]. Because it has a high Michaelis constant (K_m_), CAT remains highly functional even at extreme H_2_O_2_ concentrations [146,147]. Since the peroxisome is the center of H_2_O_2_ synthesis due to β-oxidation of fatty acids, photorespiration, oxidative stress, and purine catabolism, catalase is actively present there [148,149] and constitutes 10–25% of total peroxisomal proteins [150,151].

Glutathione peroxidase (GPx) is a crucial family of selenium-dependent antioxidant enzymes that protect cells (mainly GPx1-GPx4) from oxidative damage by reducing H_2_O_2_ and lipid hydroperoxides to water and alcohols. Working with glutathione (GSH) as a reducing agent, these enzymes mitigate toxicity, maintain redox balance, and regulate ferroptosis, a form of cell death [152,153]. There are eight types of GPx family in mammals (GPx1-GPx8), but only GPx1–4 and GPx6 in humans contain the essential amino acid selenocysteine (Sec) at their catalytic site, linking their efficiency directly to dietary selenium intake [154,155]. Most forms of GPx enzymes are found in the cytoplasm and mitochondria. Notably, GPx4 (often referred to as a Peroxidation Inhibiting Protein) plays a highly specialized role in protecting membrane lipids from oxidation, thus actively inhibiting ferroptotic cell death [156,157]. GPx deficiencies are linked to increased oxidative stress, which contributes to cardiovascular diseases, cancer, and inflammation [152,158].

Peroxiredoxin 6 (PRDX6), is a unique bifunctional enzyme with a key role in maintaining cellular homeostasis. PRDX6 is a crucial antioxidant enzyme that reduces H_2_O_2_ and repairs damaged cell membranes but it response to H_2_O_2_ depends heavily on concentration and whose function depends on the concentration of H_2_O_2_ [159]. Unlike other members of the mammalian PRDX family (which are 2-Cys enzymes requiring thioredoxin) [160,161], PRDX6 is the sole mammalian 1-Cys enzyme that does not rely on thioredoxin as a reductant [162]. It possesses two distinct catalytic activities: its peroxidase function reduces H_2_O_2_ and lipid hydroperoxides, while its acidic calcium-independent phospholipase A_2_ (aiPLA)_2_ activity repairs oxidized membranes via phospholipid hydrolysis, making it a multifunctional protector against oxidative injury. Consequently, PRDX6 acts as a fundamental biological link between neutralizing reactive oxygen species and regulating membrane dynamics, ensuring the sustained structural integrity of the epithelial cell.

Crucially, PRDX6 exhibits a dualistic mode of metabolic behavior dependent entirely on the concentration of H_2_O_2_ [159]. At physiological concentrations of H_2_O_2_ (<100 μM), it acts as an antioxidant, protecting the cell from DNA and lipid damage. However, at toxic concentrations of H_2_O_2_ (>100 μM), irreversible hyperoxidation of its critical cysteine residue (Cys47) occurs. This metabolic reversal heavily amplifies its aiPLA2 activity, leading to the strong activation of NOX2 enzymes, the generation of new ROS, cell cycle arrest in the G2/M phase, and an ultimate worsening of oxidative stress in the pathological microenvironment [162,163,164,165,166,167,168].

The robust peroxidase activity of PRDX6 underscores its systemic biological importance. Consequently, its role was studied in the etiology of numerous oxidative-driven conditions, ranging from metabolic dysfunction and cellular senescence to neurodegeneration and oncogenesis [169].

The influence of HPV on different antioxidant enzymes and their potential diagnostic or prognostic value has extensively been reviewed previously [14]; however, it warrants repeating that HPV infection was found to decrease most of the abovementioned protective enzymes in the peripheral blood of cervical cancer patients (SOD, CAT, and GPx) while PRDX6 was upregulated in cancer tissues.

## 5. Arrested Apoptosis: Failure of Tissue Homeostasis and the Biological Basis for the Progression of CIN to Invasive Carcinoma

As persistent HPV infection and chronic oxidative stress overwhelm the enzymatic antioxidant defenses, the resulting macromolecular damage—particularly to host DNA—should physiologically trigger programmed cell death. However, carcinogenesis in the cervical epithelium is fundamentally characterized by the HPV-associated dysfunction of this apoptotic regulatory mechanism, enabling the survival of damaged cells and their progression from CIN to invasive carcinoma [170].

The early indicators of this malignant progression, such as basal cell hyperplasia and dysplasia [171,172], are driven directly by the viral disruption of cellular stress sensors. Specifically, the *TP53* gene acts as the primary cellular stress sensor and the mutations of p53 protein, a well-known tumor suppressor, contribute to the development of up to 50% of all human cancers [173,174]. In stress-free cells, p53 is targeted for degradation by the E3 ubiquitin ligase MDM2, keeping wild-type p53 at low or undetectable levels [175,176]. When physiological stress signals occur, such as ROS-induced DNA damage or hypoxia, p53 is relieved from MDM2 inhibition [175,176] and directs the cell toward growth arrest or apoptosis [177].

In HPV-driven pathologies, this protective mechanism is actively dismantled. The viral oncoprotein E6 directly binds and degrades p53, while E7 targets the retinoblastoma protein (pRb), synergistically altering cell-cycle control and driving chromosomal instability [178]. In response to DNA damage, the p53 checkpoint protein safeguards genetic stability by forcing cells into either terminal apoptosis or a temporary G1 arrest [179]. This proliferative halt is executed through p53’s targeted transcriptional activation of CDKi proteins, specifically p21^WAF1/Cip1^ and p27^kip1^, thereby overriding the normal cell division cycle. P21^Waf1/Cip1^ and p27^Kip1^ are members of the Cip/Kip family, regulate the G1-S transition, are considered putative tumor suppressors, and critically both are targeted by HPV E7 [180,181]. Consequently, p53-induced apoptosis is fundamentally blocked in HPV-associated lesions, a process that can be further exacerbated by the overexpression of anti-apoptotic proteins like BCL-2 [182].

The failure of p53-mediated apoptosis directly intersects with the mitochondrial oxidative stress discussed in previous chapters. In a functional cellular response, severe ROS-induced DNA damage prompts wild-type p53 to upregulate pro-apoptotic members of the Bcl-2 family, specifically Bax and Bak [183]. These executioner proteins oligomerize to puncture the mitochondrial outer membrane, releasing cytochrome c into the cytosol and triggering the irreversible caspase cascade [184]. Cytosolic cytochrome c drives the structural formation of the apoptosome alongside APAF-1 and caspase-9, ultimately unleashing effector caspases to enzymatically dismantle the cell [185]. The targeted disruption of this p53-governed checkpoint removes a vital biological barrier to uninhibited division, acting as a ubiquitous catalyst in the development of diverse cancers [186]. In cervical cancer, because the viral E6 oncoprotein degrades p53 as well as proapoptotic Bak proteins [187], this critical communication between the nucleus and the mitochondria is severed, shielding those cells from caspase-mediated execution, allowing them to survive and accumulate the genomic instability necessary for malignant progression.

The complexity of this apoptotic failure is deepened by the involvement of the broader p53 family. Two closely related proteins, p63 [188] and p73 [189], share critical DNA-binding and transactivation architectures with p53. Consequently, these related transcription factors possess the overlapping capacity to govern canonical p53-responsive genes and their associated apoptotic cascades [190]. As evidenced by overexpression models, the independent activation of either p63 or p73 is capable of triggering cell death by apparently biochemically and morphologically classic apoptosis [188,191].

Adding further complexity to this apoptotic blockade is the paradoxical role of the p53 family member, p63. While full-length p63 (TAp63) possesses transactivation domains capable of inducing apoptosis, cervical cancers, originating as squamous cell carcinomas, characteristically overexpress a truncated isoform known as deltaNp63 [192]. Lacking the N-terminal transactivation domain, deltaNp63 functions not as a tumor suppressor, but as a potent oncogene. It acts as a dominant-negative inhibitor, competitively binding to p53 DNA response elements and actively blocking any residual wild-type p53 or related p73 from initiating cell death [193].

Ultimately, the interaction between the p53 family, HPV oncoproteins, and reactive oxygen species is not a simple linear metabolic pathway. Despite current knowledge regarding p53-mediated programmed cell death, numerous questions remain regarding the precise microenvironmental triggers that solidify this loss of apoptosis [194]. Identifying the specific oxidative conditions that finalize the transformation of CIN into invasive cancer remains a critical focus for future basic research.

## 6. Senescence-Associated Secretory Phenotype (SASP) as CIN Driver

The physiological defense against malignant transformation fundamentally depends on two distinct biological barriers to manage cells with accumulated somatic damage: apoptosis and cellular senescence [195]. While apoptosis eliminates the cell entirely, cellular senescence, originally discovered as a replicative limit in culture [196], can be simplified as a state of permanent cell cycle arrest at the G1 phase, induced by persistent DNA damage and other stress signals [197]. Nevertheless, the overarching biological implications of this arrest are highly paradoxical, as senescent cells exert distinctly dichotomous effects that can either suppress or in some cases actively facilitate tumorigenesis [24].

Senescence and cervical intraepithelial neoplasia (CIN) are deeply interconnected processes, emerging from the female body’s fight against persistent HPV infection. When viral E6 and E7 oncoproteins successfully dismantle the p53 and pRb apoptotic pathways (as discussed above), the infected cell attempts a secondary blockade. Specifically in the context of cervical precancer (CIN), it has been shown that cells infected with high-risk HPV aim to activate a senescence program (primarily via the remaining p16INK4a/pRb or stress-induced p53/p21CIP1 axes) to prevent the uncontrolled division of damaged DNA [198,199]. Senescence, while acting as a biological substitute for arrested apoptosis, is unfortunately rarely robust enough to permanently neutralize the virus.

From the perspective of a clinical gynecologist, this creates a profound physiological paradox that is often underappreciated: the normal epithelium of the ectocervix is actively remodeled by proliferation, maturation, and desquamation, completely replacing itself every 4–5 days [200]. Therefore, the clinical reality of a persistent CIN2/CIN3 lesion surviving for months or years indicates that homeostatic apoptosis has profoundly failed, and the “replacement” mechanism of senescence has taken over [201].

However, relying on stable cell cycle arrest is a flawed biological solution. As introduced earlier, senescence acts as a double-edged sword. Initially, it suppresses intraepithelial lesions by halting proliferation and enhancing immune surveillance [202,203], but if the immune system fails to clear these senescent cells, they can develop the senescence-associated secretory phenotype (SASP) and subsequently accumulate to adversely influence the tumor microenvironment (TME) [204]. SAPS was shown to have adverse impact on survival of cervical cancer patients [205].

To fully appreciate the pathological impact of SASP in cervical carcinogenesis, it is necessary to examine the specific molecular composition as well as generation of this secreted “cocktail.” Driven largely by the chronic oxidative stress detailed in previous chapters, accumulated ROS triggers SASP not only through genomic lesions (without p53 acting as a brake) but also via a potent DNA-damage-independent mechanism. Specifically, excess ROS robustly activates the p38 mitogen-activated protein kinase (MAPK) pathway. This kinase cascade persistently stimulates the transcription factor NF-kappaB, the master regulator of the pro-inflammatory SASP response [206]. Acting in tandem HPV itself triggers DNA damage responses [207], while HPV E6 oncoprotein creates a profoundly p53-deficient microenvironment. Without this natural braking mechanism, these p53-depleted senescent cells develop an further amplified secretory phenotype [208]. On the other hand, it has been noted that when E6 oncoprotein is inhibited, by in vitro E2 expression in HPV16 containing SiHa cells, the cells became more susceptible to senescence arrest and potentiate NF-kappaB signaling [209] strongly linking HPV with apopotosis, senescence, and SASP pathways in cervical tumorigenesis.

**Figure 2:**
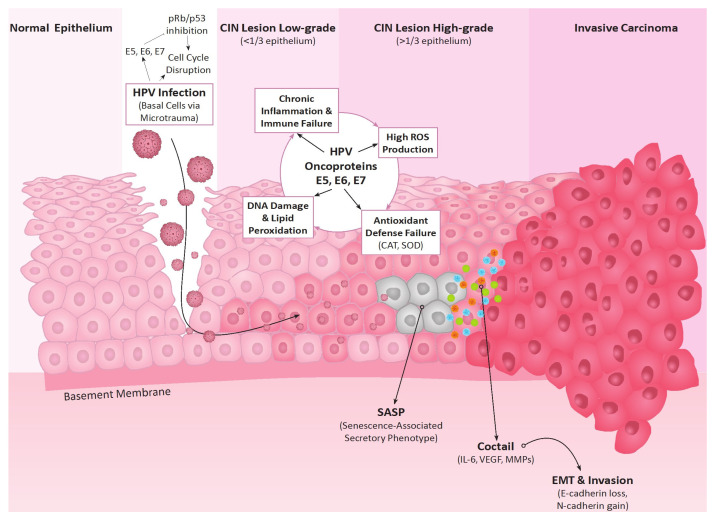
Intracellular redox imbalance and the macromolecular consequences of HPV oncoprotein expression. While the overarching oncogenic signaling cascade dictates disease progression, this figure details the specific biochemical disruptions occurring within the infected cervical epithelial cell following initial viral entry via basal epithelial microtrauma. The continuous expression of HPV oncoproteins (E5, E6, and E7), coupled with E5 potentiated mitochondrial dysfunction, generates a sustained intracellular Reactive Oxygen Species (ROS) challenge. Under normal physiological conditions, these reactive intermediates are neutralized by the cellular antioxidant defense network (i.e., SOD, CAT). However, viral-driven ROS production eventually overwhelms and exhausts this protective barrier, plunging the microenvironment into a macromolecule damaging state of chronic Oxidative Stress. Crucially, redox imbalance together with E5 triggers chronic local inflammation, which in turn acts as a secondary engine for further ROS generation, establishing a self-sustaining loop. This unmitigated stress inflicts direct, irreversible structural damage upon critical cellular macromolecules, clinically measurable as lipid peroxidation, protein carbonylation, and DNA damage. Damage leads to cellular senescence in a subset of cells which accumulate and develop Senescence-Associated Secretory Phenotype (SASP) characterized by the active release of specific pro-oncogenic effectors such as IL-6, TGF-beta. Already apoptosis resistant cells, due to HPV oncoprotein interactions, can further transform in this tumor promoting chronic inflammatory milieu by undergoing epithelial–mesenchymal transformation (EMT) and lead to invasive cancer.

Once activated, these senescent cells secrete a highly specific array of pro-tumorigenic factors into the tumor microenvironment (Figure 2). Chief among these are pro-inflammatory interleukins (particularly IL-6 and IL-8), which actively recruit immunosuppressive cells and fuel chronic inflammation [208]. Furthermore, SASP is characterized by the massive release of vascular endothelial growth factor (VEGF) to drive tumor angiogenesis, and matrix metalloproteinases (specifically MMP-1, MMP-3, and MMP-9) [208,210]. Interestingly, it has been demonstrated that HPV16 E6 is able to upregulate IL-6 expression in the SiHa and Hela Cells as well as influence cancer-associated fibroblasts within the tumor microenvironment and drive their senescence, thus directly affecting SASP [211]. Further studies highlighted other elevated SASP markers in precancerous lesions with elevated IL-8 and TGFB1 and reduced levels of MMP9 [212]. However, the secretion of these MMPs is a critical mechanical turning point in end stages of cervical cancer development: by actively degrading the extracellular matrix and the epithelial basement membrane, SASP physically clears the path for cervical intraepithelial cells to invade the underlying stroma and lead to poorer survival of cervical cancer patients [25,213].

Ultimately, the danger of SASP lies in its paracrine signaling. The localized secretion of IL-6 and TGF-beta by senescent cells does not merely sustain the inflammation of the infected cells; it actively reprograms neighboring, non-senescent epithelial cells. This paracrine bombardment forces adjacent cells to lose their apical-basal polarity and cell-to-cell adhesion, directly inducing the Epithelial–Mesenchymal Transition (EMT) and driving the final step from localized neoplasia to invasive malignancy [214].

## 7. Epithelial–Mesenchymal Transition (EMT): The Final Event in the Transformation from CIN to Invasive Carcinoma

Epithelial–mesenchymal transition (EMT) represents the ultimate biological threshold that allows cervical neoplastic cells to leave the primary lesion, breach the basement membrane, and spread to distant organs [215]. While traditionally viewed as strictly an invasive phase phenomenon, modern research demonstrates that, in the cervical cancer context, EMT is an active, measurable process driving the earlier precancerous phases of cervical intraepithelial neoplasia development (CIN) [27]. In the stepwise progression of cervical cancer, the transition is not spontaneous; it is the culmination of the pathological cascade described in previous sections. The continuous bombardment of the tissue by the senescence-associated secretory phenotype (SASP), particularly through factors like TGF-beta and IL-6, acts synergistically with viral oncoproteins and chronic oxidative stress to trigger this transformation. Under this immense microenvironmental pressure, typical polarized epithelial cells begin to dismantle their specialized adhesion complexes (tight junctions, adherens junctions, and gap junctions), losing their apical-basal polarity to become spindle-shaped, highly motile mesenchymal cells [216]. Furthermore, HPV oncoproteins were previously shown to directly influence EMT-related genes, further facilitating the transition [217,218].

Rather than an abrupt switch, this phenotypic transition can introduce a “metastable phenotype”, a hybrid cell co-expressing both epithelial and mesenchymal traits [219]. At the molecular level in cervical tissues, this phenotypic shift is driven by the gradual upregulation of key transcription factors, namely TWIST, SNAIL, and SLUG [28,220]. Activated by the ROS and SASP-rich microenvironment, these factors repress epithelial characteristics and promote mesenchymal ones, registering three distinct molecular hallmarks [220]: (1) loss of some epithelial cell markers like β-catenin and E-cadherin; (2) upregulated N-cadherin and fibronectin; (3) expression of vimentin mesenchymal intermediate filaments. Aligning with molecular biology, some EMT markers like Snail and E-cadherin were found to be prognostic in cervical cancer patients on protein [221,222] or gene levels [223].

The loss of E-cadherin is particularly devastating. As E-cadherin levels drop, the cells of the CIN lesion become structurally unstable, breaking free from their neighbors. Coupled with the matrix metalloproteinases (MMPs) secreted by the surrounding senescent cells, these newly motile, mesenchymal-like cells gain the physical capability to degrade and invade the underlying basal cell layer [28]. This gradual, molecularly driven transition from normal epithelial tissue, through HSIL, and finally into an invasive carcinoma marks the fatal completion of the HPV-induced oncogenic cycle.

## 8. New Challenges in Early Detection of Cervical Cancer

Thanks to intensive research on free radicals in biology and medicine in recent decades [67,71], we are faced with a whole spectrum of new insights into oxidative stress, aging, apoptosis, the senescence-associated secretory phenotype (SASP), and epithelial–mesenchymal transition (EMT). Redox medicine is fundamentally changing our understanding of cellular homeostasis and the pathogenesis of cancer.

Today’s clinical guidelines for managing CIN are robust, yet the incidence and mortality of cervical cancer globally highlight that current screening is not infallible. Cervical cancer must be addressed holistically—as a complex, multistep cascade of viral, oxidative, and microenvironmental events. While new research has produced a powerful array of biomarkers [224], there remains a significant translational gap between basic science and clinical medicine. The challenge is not a lack of clinical interest, but the need to standardize and integrate oxidative and microenvironmental markers into routine diagnostic protocols. Because the cervical epithelium is readily accessible for direct tissue and fluid analysis, it represents an ideal diagnostic window for these emerging panels.

### 8.1. Viral and Proliferative Biomarkers

While routine HPV DNA testing is highly sensitive, it cannot independently predict CIN evolution [225,226,227]. Emerging biomarkers offer a more dynamic picture of oncogenic activity [228] and some will be briefly mentioned here. For example, E6 and E7 mRNA levels detect active viral oncogenesis, while epigenetic markers like FAM19A4/miR124-2 methylation serve as objective risk indicators for advanced CIN3+ stages. Clinically, p16/Ki-67 dual-staining has already emerged as a highly sensitive triage tool [229]. By detecting both the surrogate marker of pRb-inactivation (p16) and cellular proliferation (Ki-67) simultaneously, it accurately identifies high-grade transforming infections and reduces unnecessary colposcopies.

### 8.2. Oxidative Stress Biomarkers

Oxidative stress biomarkers are key indicators of cellular damage and can serve as a prognostic factor in the development of CIN into invasive cancer [15,230]. The full breadth of oxidative stress biomarkers has recently been reviewed in detail [231] and only a subset relating to cervical pathology will be mentioned herein. Elevated levels of lipid oxidation by-products, notably malondialdehyde (MDA) alongside thiobarbituric acid reactive substances (TBARS), exhibit a strong positive correlation with the histopathological grading of cervical intraepithelial neoplasia [232] and are known to be increased in cancer itself [233]. However, increased MDA was suggested as prognostic for clearance of HPV infection [234] as well as complete response to chemoradiotherapy [235]. On the other hand, some studies failed to find associations with outcomes [236], or found higher preoperative MDA as associated with increased risk of recurrence and death for surgically treated oropharyngeal cancer patients, albeit with unknown HPV status [237]. Taken together, predictive potential of MDA is still not completely understood.

Beyond lipid degradation, the quantification of widespread macromolecular oxidation, specifically the mutagenic DNA adduct 8-OHdG and cervicovaginal protein carbonyls (PC), could provide predictive insight into the earliest stages of malignant progression [238], especially when paired with the measurable depletion of endogenous antioxidant enzymes like SOD, CAT, and GPx, where single-marker assessment fails to identify significant differences [236]. While 8-OHdG was also found to be elevated in cervical lesions [239,240], some authors found no differences at least for the urinary 8-OHdG measurements [48]. While not explicitly examining prognostic influence of 8-OHdG, it should be noted that cervical cancer patients demonstrated difference to urinary 8-OHdG changes post-radiotherapy compared to other cancer types, suggesting some potential for therapy response estimation [241]. Interestingly, serum protein carbonyl levels were weakly associated with genital HPV infection and possibly viral persistence in women with cervical lesions [242].

However, it needs to be mentioned that, while promising, there is still scarce data supporting clinical utility or quick implementation of OS biomarkers into therapeutic protocols in general [231]. Furthermore, it is unrealistic to expect quick implementation for cervical cancer management since historically very few biomarkers have been implemented in clinical practice in this field [243].

### 8.3. SASP and Microenvironmental Biomarkers

The detection of the SASP secretome provides a real-time snapshot of the tumor microenvironment [212,244]. Tissue and liquid biopsies can be utilized to detect significantly elevated levels of pro-inflammatory interleukins (IL-6, IL-8) and TNF-alpha, which mark the transition from protective senescence to CIN progression [245,246,247]. Furthermore, tracking the overexpression of VEGF and matrix metalloproteinases (specifically MMP-2 and MMP-9) serves as a critical early warning sign of impending basement membrane degradation and neovascularization in cervical cancer development [248,249].

### 8.4. EMT and Stemness Markers

Finally, tracking the epithelial–mesenchymal transition allows clinicians to measure the invasive potential of a lesion. The progressive upregulation of transcription factors (TWIST, SNAIL, SLUG) from CIN1 to CIN3 signals the dangerous shift toward a motile phenotype [220]. Concurrently, the standard isoform of CD44 (CD44s), a recognized cancer stem cell marker, initiates this process by driving the expression of mesenchymal markers (N-cadherin, vimentin, fibronectin) at the expense of E-cadherin [223,250,251]. Studies have shown that intense, diffuse expression of CD44 is associated with higher-grade CIN lesions and their progression to invasive carcinoma [252]. As stated above, some EMT markers like Snail and E-cadherin were found to be prognostic in cervical cancer patients [221,222,223].

## 9. Conclusions

By shifting the diagnostic paradigm to include this holistic panel of biomarkers, encompassing viral activity, oxidative damage, SASP, and EMT, we can identify high-risk lesions long before morphological invasion occurs. Ultimately, understanding these interconnected molecular networks provides more than just diagnostic accuracy; it offers profound therapeutic hope. Moving forward, targeting these specific pathways, whether through localized antioxidant therapies, EMT inhibitors, or senolytics to clear SASP-producing cells, may finally provide the tools necessary to halt the progression of cervical cancer at its source.

## Figures and Tables

**Table 1:** Critical reactive oxygen species (ROS) involved in signaling and pathology.

ROS	Half-Life t_1/2_ (s)	Migration Distance	Reactivity
Superoxide anion (O_2_^•−^)	~10^−6^	~30 nm	Selective
Hydrogen peroxide (H_2_O_2_)	~10^−3^	>1 μm	Selective
Hydroxyl radical (^•^OH)	~10^−9^	~1 nm	Indiscriminate damage

## Data Availability

No new data were created or analyzed in this study. Data sharing is not applicable to this article.

## Abbreviations

The following abbreviations are used in this manuscript:

HPV	Human papillomavirus
OS	Oxidative stress
SASP	Senescence associated secretory phenotype
EMT	epithelial–mesenchymal transition
CIN	Cervical intraepithelial neoplasia
CIN 1	Low grade cervical intraepithelial neoplasia
CIN 2	Moderate grade cervical intraepithelial neoplasia
CIN 3	Severe cervical intraepithelial neoplasia
LSIL	Low grade squamous epithelial lesion
HSIL	High grade squamous epithelial lesion
CC	Cervical cancer
WHO	World Health Organization
IARC	the International Agency for Research on Cancer
ROS	reactive oxygen species
MMPs	Matrix metalloproteinases

## References

[1] Bray F., Laversanne M., Sung H., Ferlay J., Siegel R.L., Soerjomataram I., Jemal A. (2024). Global Cancer Statistics 2022: GLOBOCAN Estimates of Incidence and Mortality Worldwide for 36 Cancers in 185 Countries. CA Cancer J. Clin..

[2] Okunade K.S. (2020). Human Papillomavirus and Cervical Cancer. J. Obstet. Gynaecol. J. Inst. Obstet. Gynaecol..

[3] Kusakabe M., Taguchi A., Sone K., Mori M., Osuga Y. (2023). Carcinogenesis and Management of Human Papillomavirus-Associated Cervical Cancer. Int. J. Clin. Oncol..

[4] Lowy D.R. (2024). Harald Zur Hausen (1936 to 2023): Discoverer of Human Papillomavirus Infection as the Main Cause of Cervical Cancer. Proc. Natl. Acad. Sci. USA.

[5] Vats A., Trejo-Cerro O., Thomas M., Banks L. (2021). Human Papillomavirus E6 and E7: What Remains?. Tumour Virus Res..

[6] Bergqvist L., Virtanen A., Kalliala I., Bützow R., Jakobsson M., Heinonen A., Louvanto K., Dillner J., Nieminen P., Aro K. (2025). Predictors for Regression and Progression of Actively Surveilled Cervical Intraepithelial Neoplasia Grade 2: A Prospective Cohort Study. Acta Obstet. Gynecol. Scand..

[7] Tainio K., Athanasiou A., Tikkinen K.A.O., Aaltonen R., Cárdenas Hernándes J., Glazer-Livson S., Jakobsson M., Joronen K., Kiviharju M., Louvanto K. (2018). Clinical Course of Untreated Cervical Intraepithelial Neoplasia Grade 2 under Active Surveillance: Systematic Review and Meta-Analysis. BMJ.

[8] Loopik D.L., IntHout J., Ebisch R.M.F., Melchers W.J.G., Massuger L.F.A.G., Siebers A.G., Bekkers R.L.M. (2020). The Risk of Cervical Cancer after Cervical Intraepithelial Neoplasia Grade 3: A Population-Based Cohort Study with 80,442 Women. Gynecol. Oncol..

[9] World Health Organization (2020). Cervical cancer elimination initiative. Global Strategy to Accelerate the Elimination of Cervical Cancer as a Public Health Problem.

[10] Pizzino G., Irrera N., Cucinotta M., Pallio G., Mannino F., Arcoraci V., Squadrito F., Altavilla D., Bitto A. (2017). Oxidative Stress: Harms and Benefits for Human Health. Oxid. Med. Cell. Longev..

[11] Abele D. (2002). Toxic Oxygen: The Radical Life-Giver. Nature.

[12] Hong Y., Boiti A., Vallone D., Foulkes N.S. (2024). Reactive Oxygen Species Signaling and Oxidative Stress: Transcriptional Regulation and Evolution. Antioxidants.

[13] Letafati A., Taghiabadi Z., Zafarian N., Tajdini R., Mondeali M., Aboofazeli A., Chichiarelli S., Saso L., Jazayeri S.M. (2024). Emerging Paradigms: Unmasking the Role of Oxidative Stress in HPV-Induced Carcinogenesis. Infect. Agent. Cancer.

[14] Preci D.P., Almeida A., Weiler A.L., Franciosi M.L.M., Cardoso A.M. (2021). Oxidative Damage and Antioxidants in Cervical Cancer. Int. J. Gynecol. Cancer.

[15] Anton E.T.-T., Anton G.-I., Scripcariu I.-S., Dumitrașcu I., Scripcariu D.V., Balmus I.-M., Ionescu C., Visternicu M., Socolov D.G. (2025). Oxidative Stress, Inflammation, and Antioxidant Strategies in Cervical Cancer—A Narrative Review. Int. J. Mol. Sci..

[16] Mahdi M.A., Dawood Y.J., Jumaa A.H., Abod K.S., Manssor K.J. (2026). From ROS to Tumorigenesis: Understanding the Oxidative Pathways in Cervical Cancer Progression. Asian Pac. J. Cancer Biol..

[17] Wang H., Guo M., Wei H., Chen Y. (2023). Targeting P53 Pathways: Mechanisms, Structures and Advances in Therapy. Signal Transduct. Target. Ther..

[18] Zuckerman V., Wolyniec K., Sionov R.V., Haupt S., Haupt Y. (2009). Tumour Suppression by P53: The Importance of Apoptosis and Cellular Senescence. J. Pathol..

[19] Schmitt C.A., Fridman J.S., Yang M., Lee S., Baranov E., Hoffman R.M., Lowe S.W. (2002). A Senescence Program Controlled by P53 and p16INK4a Contributes to the Outcome of Cancer Therapy. Cell.

[20] LaPak K.M., Burd C.E. (2014). The Molecular Balancing Act of P16(INK4a) in Cancer and Aging. Mol. Cancer Res. MCR.

[21] DeFilippis R.A., Goodwin E.C., Wu L., DiMaio D. (2003). Endogenous Human Papillomavirus E6 and E7 Proteins Differentially Regulate Proliferation, Senescence, and Apoptosis in HeLa Cervical Carcinoma Cells. J. Virol..

[22] Horner S.M., DeFilippis R.A., Manuelidis L., DiMaio D. (2004). Repression of the Human Papillomavirus E6 Gene Initiates P53-Dependent, Telomerase-Independent Senescence and Apoptosis in HeLa Cervical Carcinoma Cells. J. Virol..

[23] Sima N., Wang S., Wang W., Kong D., Xu Q., Tian X., Luo A., Zhou J., Xu G., Meng L. (2007). Antisense Targeting Human Papillomavirus Type 16 E6 and E7 Genes Contributes to Apoptosis and Senescence in SiHa Cervical Carcinoma Cells. Gynecol. Oncol..

[24] Feng T., Xie F., Lee L.M.Y., Lin Z., Tu Y., Lyu Y., Yu P., Wu J., Chen B., Zhang G. (2025). Cellular Senescence in Cancer: From Mechanism Paradoxes to Precision Therapeutics. Mol. Cancer.

[25] Purohit S., Zhi W., Ferris D.G., Alverez M., Tran L.K.H., Tran P.M.H., Dun B., Hopkins D., dos Santos B., Ghamande S. (2020). Senescence-Associated Secretory Phenotype Determines Survival and Therapeutic Response in Cervical Cancer. Cancers.

[26] Kalluri R., Weinberg R.A. (2009). The Basics of Epithelial-Mesenchymal Transition. J. Clin. Investig..

[27] Lee M.-Y., Shen M.-R. (2012). Epithelial-Mesenchymal Transition in Cervical Carcinoma. Am. J. Transl. Res..

[28] Popiel-Kopaczyk A., Piotrowska A., Sputa-Grzegrzolka P., Smolarz B., Romanowicz H., Dziegiel P., Podhorska-Okolow M., Kobierzycki C. (2023). The Immunohistochemical Expression of Epithelial–Mesenchymal Transition Markers in Precancerous Lesions and Cervical Cancer. Int. J. Mol. Sci..

[29] Meyer M., Fourie C., van der Merwe H., Botha H., Engelbrecht A.-M. (2025). Targeting Treatment Resistance in Cervical Cancer: A New Avenue for Senolytic Therapies. Adv. Med. Sci..

[30] Cruz-Gregorio A., Aranda-Rivera A.K., Ortega-Lozano A.J., Pedraza-Chaverri J., Mendoza-Hoffmann F. (2021). Lipid Metabolism and Oxidative Stress in HPV-Related Cancers. Free Radic. Biol. Med..

[31] Cruz-Gregorio A., Aranda-Rivera A.K., Roviello G.N., Pedraza-Chaverri J. (2023). Targeting Mitochondrial Therapy in the Regulation of HPV Infection and HPV-Related Cancers. Pathogens.

[32] Shao Q., Liu T., Hu B., Chen L. (2025). Interplay between Autophagy and Apoptosis in Human Viral Pathogenesis. Virus Res..

[33] Yadav C., Yadav R., Chabbra R., Nanda S., Ranga S., Kadian L., Ahuja P. (2023). Overview of Genetic and Epigenetic Regulation of Human Papillomavirus and Apoptosis in Cervical Cancer. Apoptosis Int. J. Program. Cell Death.

[34] Liu Y., Zhang Y., Liu W. (2025). Cytoskeletal Remodeling by Oncoviruses: A Key Factor in Tumor Invasion and Metastasisa. Cell Biochem. Funct..

[35] Barillari G., Bei R., Manzari V., Modesti A. (2021). Infection by High-Risk Human Papillomaviruses, Epithelial-to-Mesenchymal Transition and Squamous Pre-Malignant or Malignant Lesions of the Uterine Cervix: A Series of Chained Events?. Int. J. Mol. Sci..

[36] Bzhalava D., Eklund C., Dillner J. (2015). International Standardization and Classification of Human Papillomavirus Types. Virology.

[37] Van Doorslaer K., Li Z., Xirasagar S., Maes P., Kaminsky D., Liou D., Sun Q., Kaur R., Huyen Y., McBride A.A. (2017). The Papillomavirus Episteme: A Major Update to the Papillomavirus Sequence Database. Nucleic Acids Res..

[38] Muñoz N., Bosch F.X., de Sanjose S., Herrero R., Castellsague X., Shah K.V., Snijders P.J.F., Meijer C.J. (2003). IARC Study Group. Epidemiologic Classification of Human Papillomavirus Types Associated with Cervical Cancer. N. Engl. J. Med..

[39] Mistry N., Wibom C., Evander M. (2008). Cutaneous and Mucosal Human Papillomaviruses Differ in Net Surface Charge, Potential Impact on Tropism. Virol. J..

[40] Prendiville W., Sankaranarayanan R. (2017). Anatomy of the Uterine Cervix and the Transformation Zone. Colposcopy and Treatment of Cervical Precancer.

[41] Cosper P.F., Bradley S., Luo L., Kimple R.J. (2021). Biology of HPV Mediated Carcinogenesis and Tumor Progression. Semin. Radiat. Oncol..

[42] Burd E.M. (2003). Human Papillomavirus and Cervical Cancer. Clin. Microbiol. Rev..

[43] Doorbar J., Griffin H. (2019). Refining Our Understanding of Cervical Neoplasia and Its Cellular Origins. Papillomavirus Res..

[44] de Martel C., Georges D., Bray F., Ferlay J., Clifford G.M. (2020). Global Burden of Cancer Attributable to Infections in 2018: A Worldwide Incidence Analysis. Lancet Glob. Health.

[45] Schiffman M., Doorbar J., Wentzensen N., de Sanjosé S., Fakhry C., Monk B.J., Stanley M.A., Franceschi S. (2016). Carcinogenic Human Papillomavirus Infection. Nat. Rev. Dis. Primer.

[46] Cruz-Gregorio A., Manzo-Merino J., Lizano M. (2018). Cellular Redox, Cancer and Human Papillomavirus. Virus Res..

[47] Georgescu S.R., Mitran C.I., Mitran M.I., Caruntu C., Sarbu M.I., Matei C., Nicolae I., Tocut S.M., Popa M.I., Tampa M. (2018). New Insights in the Pathogenesis of HPV Infection and the Associated Carcinogenic Processes: The Role of Chronic Inflammation and Oxidative Stress. J. Immunol. Res..

[48] Looi M.-L., Mohd Dali A.Z.H., Md Ali S.A., Wan Ngah W.Z., Mohd Yusof Y.A. (2008). Oxidative Damage and Antioxidant Status in Patients with Cervical Intraepithelial Neoplasia and Carcinoma of the Cervix. Eur. J. Cancer Prev. Off. J. Eur. Cancer Prev. Organ..

[49] Gore M., Kabekkodu S.P., Chakrabarty S. (2025). Exploring the Metabolic Alterations in Cervical Cancer Induced by HPV Oncoproteins: From Mechanisms to Therapeutic Targets. Biochim. Biophys. Acta Rev. Cancer.

[50] Tsoneva E., Yordanov A. (2025). HPV Oncoproteins and Mitochondrial Reprogramming: The Central Role of ROMO1 in Oxidative Stress and Metabolic Shifts. Cells.

[51] Basukala O., Banks L. (2021). The Not-So-Good, the Bad and the Ugly: HPV E5, E6 and E7 Oncoproteins in the Orchestration of Carcinogenesis. Viruses.

[52] Ilahi N.E., Bhatti A. (2020). Impact of HPV E5 on Viral Life Cycle via EGFR Signaling. Microb. Pathog..

[53] Scarth J.A., Patterson M.R., Morgan E.L., Macdonald A. (2021). The Human Papillomavirus Oncoproteins: A Review of the Host Pathways Targeted on the Road to Transformation. J. Gen. Virol..

[54] Campo M.S., Graham S.V., Cortese M.S., Ashrafi G.H., Araibi E.H., Dornan E.S., Miners K., Nunes C., Man S. (2010). HPV-16 E5 down-Regulates Expression of Surface HLA Class I and Reduces Recognition by CD8 T Cells. Virology.

[55] Hemmat N., Bannazadeh Baghi H. (2019). Association of Human Papillomavirus Infection and Inflammation in Cervical Cancer. Pathog. Dis..

[56] Fernandes J.V., DE Medeiros Fernandes T.A.A., DE Azevedo J.C.V., Cobucci R.N.O., DE Carvalho M.G.F., Andrade V.S., DE Araújo J.M.G. (2015). Link between Chronic Inflammation and Human Papillomavirus-Induced Carcinogenesis (Review). Oncol. Lett..

[57] Hoellen F., Waldmann A., Banz-Jansen C., Rody A., Heide M., Köster F., Ribbat-Idel J., Thorns C., Gebhard M., Oberländer M. (2016). Expression of Cyclooxygenase-2 in Cervical Cancer Is Associated with Lymphovascular Invasion. Oncol. Lett..

[58] Li Q., Kaidong L., Tian Z., Diao W., Sun Y., Bai Y., Ma Y., Wei Y., Li J., Zhao W. (2024). Association of Inflammatory Factors with Cervical Cancer: A Bidirectional Mendelian Randomization. J. Inflamm. Res..

[59] Kumar S., Biswas M., Jose T. (2015). HPV Vaccine: Current Status and Future Directions. Med. J. Armed Forces India.

[60] Doorbar J. (2023). The Human Papillomavirus Twilight Zone—Latency, Immune Control and Subclinical Infection. Tumour Virus Res..

[61] Lynge E., Lönnberg S., Törnberg S. (2017). Cervical Cancer Incidence in Elderly Women-Biology or Screening History?. Eur. J. Cancer.

[62] Quick A.M., Krok-Schoen J.L., Stephens J.A., Fisher J.L. (2020). Cervical Cancer Among Older Women: Analyses of Surveillance, Epidemiology and End Results Program Data. Cancer Control J. Moffitt Cancer Cent..

[63] Crum C.P., McLachlin C.M. (1995). Cervical Intraepithelial Neoplasia. J. Cell. Biochem. Suppl..

[64] Ostör A.G. (1993). Natural History of Cervical Intraepithelial Neoplasia: A Critical Review. Int. J. Gynecol. Pathol. Off. J. Int. Soc. Gynecol. Pathol..

[65] Vink M.A., Bogaards J.A., van Kemenade F.J., de Melker H.E., Meijer C.J.L.M., Berkhof J. (2013). Clinical Progression of High-Grade Cervical Intraepithelial Neoplasia: Estimating the Time to Preclinical Cervical Cancer from Doubly Censored National Registry Data. Am. J. Epidemiol..

[66] Bedell S.L., Goldstein L.S., Goldstein A.R., Goldstein A.T. (2020). Cervical Cancer Screening: Past, Present, and Future. Sex. Med. Rev..

[67] Hopkins R., Li Y.R. (2017). Essentials of Free Radical Biology and Medicine.

[68] Schieber M., Chandel N.S. (2014). ROS Function in Redox Signaling and Oxidative Stress. Curr. Biol. CB.

[69] Andrés C.M.C., Pérez de la Lastra J.M., Andrés Juan C., Plou F.J., Pérez-Lebeña E. (2023). Superoxide Anion Chemistry—Its Role at the Core of the Innate Immunity. Int. J. Mol. Sci..

[70] Nandi A., Yan L.-J., Jana C.K., Das N. (2019). Role of Catalase in Oxidative Stress- and Age-Associated Degenerative Diseases. Oxid. Med. Cell. Longev..

[71] Halliwell B., Gutteridge J.M.C. (2015). Free Radicals in Biology and Medicine.

[72] Hayes J.D., Dinkova-Kostova A.T., Tew K.D. (2020). Oxidative Stress in Cancer. Cancer Cell.

[73] Arfin S., Jha N.K., Jha S.K., Kesari K.K., Ruokolainen J., Roychoudhury S., Rathi B., Kumar D. (2021). Oxidative Stress in Cancer Cell Metabolism. Antioxidants.

[74] Dumanović J., Nepovimova E., Natić M., Kuča K., Jaćević V. (2021). The Significance of Reactive Oxygen Species and Antioxidant Defense System in Plants: A Concise Overview. Front. Plant Sci..

[75] Hayyan M., Hashim M.A., AlNashef I.M. (2016). Superoxide Ion: Generation and Chemical Implications. Chem. Rev..

[76] Sawyer D.T., Valentine J.S. (1981). How Super Is Superoxide?. Acc. Chem. Res..

[77] Hayyan M., Mjalli F.S., AlNashef I.M., Hashim M.A. (2012). Chemical and Electrochemical Generation of Superoxide Ion in 1-Butyl-1-Methylpyrrolidinium Bis(Trifluoromethylsulfonyl)Imide. Int. J. Electrochem. Sci..

[78] Carmo De Carvalho E Martins M.D., Martins, Da Silva Santos Oliveira A.S., Da Silva L.A.A., Primo M.G.S., De Carvalho Lira V.B., Patel V.B., Preedy V.R. (2022). Biological Indicators of Oxidative Stress [Malondialdehyde, Catalase, Glutathione Peroxidase, and Superoxide Dismutase] and Their Application in Nutrition. Biomarkers in Nutrition.

[79] Alberts B., Johnson A., Lewis J., Morgan D., Raff M., Roberts K., Walter P. (2015). Energy Conversion: Mitochondria and Chloroplasts. Molecular Biology of the Cell.

[80] Fridovich I. (2013). Oxygen: How Do We Stand It?. Med. Princ. Pract. Int. J. Kuwait Univ. Health Sci. Cent..

[81] Mimaki M., Wang X., McKenzie M., Thorburn D.R., Ryan M.T. (2012). Understanding Mitochondrial Complex I Assembly in Health and Disease. Biochim. Biophys. Acta.

[82] Fridovich I. (1999). Fundamental Aspects of Reactive Oxygen Species, or What’s the Matter with Oxygen?. Ann. N. Y. Acad. Sci..

[83] Murphy M.P. (2009). How Mitochondria Produce Reactive Oxygen Species. Biochem. J..

[84] Brand M.D. (2016). Mitochondrial Generation of Superoxide and Hydrogen Peroxide as the Source of Mitochondrial Redox Signaling. Free Radic. Biol. Med..

[85] Quinlan C.L., Perevoshchikova I.V., Hey-Mogensen M., Orr A.L., Brand M.D. (2013). Sites of Reactive Oxygen Species Generation by Mitochondria Oxidizing Different Substrates. Redox Biol..

[86] Muller F.L., Liu Y., Van Remmen H. (2004). Complex III Releases Superoxide to Both Sides of the Inner Mitochondrial Membrane. J. Biol. Chem..

[87] Stoolman J.S., Grant R.A., Billingham L.K., Poor T.A., Weinberg S.E., Harding M.C., Lu Z., Miska J., Szibor M., Budinger G.S. (2025). Mitochondria Complex III–Generated Superoxide Is Essential for IL-10 Secretion in Macrophages. Sci. Adv..

[88] Vermot A., Petit-Härtlein I., Smith S.M.E., Fieschi F. (2021). NADPH Oxidases (NOX): An Overview from Discovery, Molecular Mechanisms to Physiology and Pathology. Antioxidants.

[89] Cipriano A., Viviano M., Feoli A., Milite C., Sarno G., Castellano S., Sbardella G. (2023). NADPH Oxidases: From Molecular Mechanisms to Current Inhibitors. J. Med. Chem..

[90] Hille R. (2023). Xanthine Oxidase-A Personal History. Molecules.

[91] Bortolotti M., Polito L., Battelli M.G., Bolognesi A. (2021). Xanthine Oxidoreductase: One Enzyme for Multiple Physiological Tasks. Redox Biol..

[92] Aziz N., Jamil R.T. (2025). Biochemistry, Xanthine Oxidase. StatPearls.

[93] Cruz-Gregorio A., Manzo-Merino J., Gonzaléz-García M.C., Pedraza-Chaverri J., Medina-Campos O.N., Valverde M., Rojas E., Rodríguez-Sastre M.A., García-Cuellar C.M., Lizano M. (2018). Human Papillomavirus Types 16 and 18 Early-Expressed Proteins Differentially Modulate the Cellular Redox State and DNA Damage. Int. J. Biol. Sci..

[94] Marullo R., Werner E., Zhang H., Chen G.Z., Shin D.M., Doetsch P.W. (2015). HPV16 E6 and E7 Proteins Induce a Chronic Oxidative Stress Response via NOX2 That Causes Genomic Instability and Increased Susceptibility to DNA Damage in Head and Neck Cancer Cells. Carcinogenesis.

[95] Lai D., Tan C.L., Gunaratne J., Quek L.S., Nei W., Thierry F., Bellanger S. (2013). Localization of HPV-18 E2 at Mitochondrial Membranes Induces ROS Release and Modulates Host Cell Metabolism. PLoS ONE.

[96] Bianchini B.H.S., Martelossi-Cebinelli G., Carneiro J.A., Rasquel-Oliveira F.S., Casagrande R., Verri W.A. (2025). Superoxide Anion Generation, Its Pathological Cellular and Molecular Roles and Pharmacological Targeting in Inflammatory Pain: Lessons from the Potassium Superoxide Model. Future Pharmacol..

[97] Fukai T., Ushio-Fukai M. (2011). Superoxide Dismutases: Role in Redox Signaling, Vascular Function, and Diseases. Antioxid. Redox Signal..

[98] Sies H. (2017). Hydrogen Peroxide as a Central Redox Signaling Molecule in Physiological Oxidative Stress: Oxidative Eustress. Redox Biol..

[99] Koppenol W.H., Sies H. (2024). Was Hydrogen Peroxide Present before the Arrival of Oxygenic Photosynthesis? The Important Role of Iron(II) in the Archean Ocean. Redox Biol..

[100] Saxena I., Srikanth S., Chen Z. (2016). Cross Talk between H_2_O_2_ and Interacting Signal Molecules under Plant Stress Response. Front. Plant Sci..

[101] Park S., Kim C., Heo S., Kang D. (2025). Endosomal H2O2 Molecules Act as Signaling Mediators in Akt/PKB Activation. Antioxidants.

[102] Marinho H.S., Real C., Cyrne L., Soares H., Antunes F. (2014). Hydrogen Peroxide Sensing, Signaling and Regulation of Transcription Factors. Redox Biol..

[103] Imlay J.A. (2015). Transcription Factors That Defend Bacteria Against Reactive Oxygen Species. Annu. Rev. Microbiol..

[104] Sun C., Diao H., Zhang X., Han Z., Wei J. (2025). Study on Human Papillomavirus (HPV) Infection, Typing, Peripheral Blood Immune Factor Expression and Vaginal Microenvironment Balance in Cervical Cancer Screening Population. Indian J. Med. Microbiol..

[105] Andrés C.M.C., Pérez de la Lastra J.M., Juan C.A., Plou F.J., Pérez-Lebeña E. (2022). Chemistry of Hydrogen Peroxide Formation and Elimination in Mammalian Cells, and Its Role in Various Pathologies. Stresses.

[106] Forman H.J., Maiorino M., Ursini F. (2010). Signaling Functions of Reactive Oxygen Species. Biochemistry.

[107] Pravda J. (2020). Hydrogen Peroxide and Disease: Towards a Unified System of Pathogenesis and Therapeutics. Mol. Med..

[108] Miller E.W., Dickinson B.C., Chang C.J. (2010). Aquaporin-3 Mediates Hydrogen Peroxide Uptake to Regulate Downstream Intracellular Signaling. Proc. Natl. Acad. Sci. USA.

[109] Bienert G.P., Chaumont F. (2014). Aquaporin-Facilitated Transmembrane Diffusion of Hydrogen Peroxide. Biochim. Biophys. Acta.

[110] Bertolotti M., Farinelli G., Galli M., Aiuti A., Sitia R. (2016). AQP8 Transports NOX_2_-Generated H_2_O_2_ across the Plasma Membrane to Promote Signaling in B Cells. J. Leukoc. Biol..

[111] Hara-Chikuma M., Watanabe S., Satooka H. (2016). Involvement of Aquaporin-3 in Epidermal Growth Factor Receptor Signaling via Hydrogen Peroxide Transport in Cancer Cells. Biochem. Biophys. Res. Commun..

[112] Rampon C., Volovitch M., Joliot A., Vriz S. (2018). Hydrogen Peroxide and Redox Regulation of Developments. Antioxidants.

[113] Fenton H.J.H. (1894). LXXIII.—Oxidation of Tartaric Acid in Presence of Iron. J. Chem. Soc. Trans..

[114] Barbusiński K. (2009). Fenton Reaction—Controversy Concerning the Chemistry. Ecol. Chem. Eng.-Chem. Inzynieria Ekol. S.

[115] Sies H. (2014). Role of Metabolic H2O2 Generation: Redox Signaling and Oxidative Stress. J. Biol. Chem..

[116] Di Marzo N., Chisci E., Giovannoni R. (2018). The Role of Hydrogen Peroxide in Redox-Dependent Signaling: Homeostatic and Pathological Responses in Mammalian Cells. Cells.

[117] Chandimali N., Bak S.G., Park E.H., Lim H.-J., Won Y.-S., Kim E.-K., Park S.-I., Lee S.J. (2025). Free Radicals and Their Impact on Health and Antioxidant Defenses: A Review. Cell Death Discov..

[118] Abe C., Miyazawa T., Miyazawa T. (2022). Current Use of Fenton Reaction in Drugs and Food. Molecules.

[119] Ali T., Li D., Ponnamperumage T.N.F., Peterson A.K., Pandey J., Fatima K., Brzezinski J., Jakusz J.A.R., Gao H., Koelsch G.E. (2024). Generation of Hydrogen Peroxide in Cancer Cells: Advancing Therapeutic Approaches for Cancer Treatment. Cancers.

[120] Stanbury D.M. (2022). The Principle of Detailed Balancing, the Iron-Catalyzed Disproportionation of Hydrogen Peroxide, and the Fenton Reaction. Dalton Trans..

[121] Lu H.-F., Chen H.-F., Kao C.-L., Chao I., Chen H.-Y. (2018). A Computational Study of the Fenton Reaction in Different pH Ranges. Phys. Chem. Chem. Phys..

[122] Kremer M.L. (2003). The Fenton Reaction. Dependence of the Rate on pH. J. Phys. Chem. A.

[123] Chen H.-Y. (2019). Why the Reactive Oxygen Species of the Fenton Reaction Switches from Oxoiron(IV) Species to Hydroxyl Radical in Phosphate Buffer Solutions? A Computational Rationale. ACS Omega.

[124] Ou R., Ai J. (2023). Advancements in the Application of the Fenton Reaction in the Cancer Microenvironment. Pharmaceutics.

[125] Vartanian A.A., Kosorukov V.S. (2025). Pro-Inflammatory Cytokines, Ferroptosis, and Cancer. Acta Naturae.

[126] Siegel E.M., Patel N., Lu B., Lee J.-H., Nyitray A.G., Huang X., Villa L.L., Franco E.L., Giuliano A.R. (2012). Circulating Biomarkers of Iron Storage and Clearance of Incident Human Papillomavirus Infection. Cancer Epidemiol. Biomark. Prev. Publ. Am. Assoc. Cancer Res. Cosponsored Am. Soc. Prev. Oncol..

[127] Chen X., Chen H., Chen Y., Tang L., Liu J., Ou Y.-H. (2025). L-Shaped Association between Dietary Iron Intake and HPV Infection: A Cross-Sectional Analysis Based on National Health and Nutrition Examination Survey 2005–2016. Front. Nutr..

[128] Rodwell V.W., Rodwell V.W., Bender D.A., Botham K.M., Kennelly P.J., Weil P.A. (2018). Conversion of Amino Acids to Specialized Products. Harper’s Illustrated Biochemistry.

[129] Thomas C., Wurzer L., Malle E., Ristow M., Madreiter-Sokolowski C.T. (2022). Modulation of Reactive Oxygen Species Homeostasis as a Pleiotropic Effect of Commonly Used Drugs. Front. Aging.

[130] Manful C.F., Fordjour E., Subramaniam D., Sey A.A., Abbey L., Thomas R. (2025). Antioxidants and Reactive Oxygen Species: Shaping Human Health and Disease Outcomes. Int. J. Mol. Sci..

[131] Jomova K., Alomar S.Y., Alwasel S.H., Nepovimova E., Kuca K., Valko M. (2024). Several Lines of Antioxidant Defense against Oxidative Stress: Antioxidant Enzymes, Nanomaterials with Multiple Enzyme-Mimicking Activities, and Low-Molecular-Weight Antioxidants. Arch. Toxicol..

[132] Ighodaro O.M., Akinloye O.A. (2018). First Line Defence Antioxidants-Superoxide Dismutase (SOD), Catalase (CAT) and Glutathione Peroxidase (GPX): Their Fundamental Role in the Entire Antioxidant Defence Grid. Alex. J. Med..

[133] Eddaikra A., Eddaikra N., Eddaikra A., Eddaikra N. (2021). Endogenous Enzymatic Antioxidant Defense and Pathologies. Antioxidants—Benefits, Sources, Mechanisms of Action.

[134] Zheng M., Liu Y., Zhang G., Yang Z., Xu W., Chen Q. (2023). The Applications and Mechanisms of Superoxide Dismutase in Medicine, Food, and Cosmetics. Antioxidants.

[135] Zelko I.N., Mariani T.J., Folz R.J. (2002). Superoxide Dismutase Multigene Family: A Comparison of the CuZn-SOD (SOD1), Mn-SOD (SOD2), and EC-SOD (SOD3) Gene Structures, Evolution, and Expression. Free Radic. Biol. Med..

[136] Del Maestro R., McDonald W. (1989). Subcellular Localization of Superoxide Dismutases, Glutathione Peroxidase and Catalase in Developing Rat Cerebral Cortex. Mech. Ageing Dev..

[137] Wu C., Xu D., Ge M., Luo J., Chen L., Chen Z., You Y., Zhu Y., Lin H., Shi J. (2022). Blocking Glutathione Regeneration: Inorganic NADPH Oxidase Nanozyme Catalyst Potentiates Tumoral Ferroptosis. Nano Today.

[138] Fisher A.B. (2009). Redox Signaling across Cell Membranes. Antioxid. Redox Signal..

[139] Rasheed Z. (2024). Therapeutic Potentials of Catalase: Mechanisms, Applications, and Future Perspectives. Int. J. Health Sci..

[140] Baker A., Lin C.-C., Lett C., Karpinska B., Wright M.H., Foyer C.H. (2023). Catalase: A Critical Node in the Regulation of Cell Fate. Free Radic. Biol. Med..

[141] Alhumaydhi F.A., Younus H., Khan M.A. (2025). Catalase Functions and Glycation: Their Central Roles in Oxidative Stress, Metabolic Disorders, and Neurodegeneration. Catalysts.

[142] Kirkman H.N., Gaetani G.F. (1984). Catalase: A Tetrameric Enzyme with Four Tightly Bound Molecules of NADPH. Proc. Natl. Acad. Sci. USA.

[143] Deisseroth A., Dounce A.L. (1970). Catalase: Physical and Chemical Properties, Mechanism of Catalysis, and Physiological Role. Physiol. Rev..

[144] Lardinois O.M. (1995). Reactions of Bovine Liver Catalase with Superoxide Radicals and Hydrogen Peroxide. Free Radic. Res..

[145] Chelikani P., Fita I., Loewen P.C. (2004). Diversity of Structures and Properties among Catalases. Cell. Mol. Life Sci. CMLS.

[146] Ogura Y. (1955). Catalase Activity at High Concentration of Hydrogen Peroxide. Arch. Biochem. Biophys..

[147] Ogura Y., Yamazaki I. (1983). Steady-State Kinetics of the Catalase Reaction in the Presence of Cyanide. J. Biochem..

[148] Mittler R. (2002). Oxidative Stress, Antioxidants and Stress Tolerance. Trends Plant Sci..

[149] Koepke J.I., Nakrieko K.-A., Wood C.S., Boucher K.K., Terlecky L.J., Walton P.A., Terlecky S.R. (2007). Restoration of Peroxisomal Catalase Import in a Model of Human Cellular Aging. Traffic.

[150] Anwar S., Alrumaihi F., Sarwar T., Babiker A.Y., Khan A.A., Prabhu S.V., Rahmani A.H. (2024). Exploring Therapeutic Potential of Catalase: Strategies in Disease Prevention and Management. Biomolecules.

[151] Al-Hajaya Y., Karpinska B., Foyer C.H., Baker A. (2022). Nuclear and Peroxisomal Targeting of Catalase. Plant Cell Environ..

[152] Handy D.E., Loscalzo J. (2022). The Role of Glutathione Peroxidase-1 in Health and Disease. Free Radic. Biol. Med..

[153] Pei J., Pan X., Wei G., Hua Y. (2023). Research Progress of Glutathione Peroxidase Family (GPX) in Redoxidation. Front. Pharmacol..

[154] Brigelius-Flohé R., Maiorino M. (2013). Glutathione Peroxidases. Biochim. Biophys. Acta.

[155] Barbosa N.V., Nogueira C.W., Nogara P.A., de Bem A.F., Aschner M., Rocha J.B.T. (2017). Organoselenium Compounds as Mimics of Selenoproteins and Thiol Modifier Agents. Metallomics.

[156] Bersuker K., Hendricks J.M., Li Z., Magtanong L., Ford B., Tang P.H., Roberts M.A., Tong B., Maimone T.J., Zoncu R. (2019). The CoQ Oxidoreductase FSP1 Acts Parallel to GPX4 to Inhibit Ferroptosis. Nature.

[157] Zhang W., Liu Y., Liao Y., Zhu C., Zou Z. (2024). GPX4, Ferroptosis, and Diseases. Biomed. Pharmacother..

[158] Lubos E., Kelly N.J., Oldebeken S.R., Leopold J.A., Zhang Y.-Y., Loscalzo J., Handy D.E. (2011). Glutathione Peroxidase-1 Deficiency Augments Proinflammatory Cytokine-Induced Redox Signaling and Human Endothelial Cell Activation. J. Biol. Chem..

[159] Kim S.Y., Jo H.-Y., Kim M.H., Cha Y., Choi S.W., Shim J.-H., Kim T.J., Lee K.-Y. (2008). H2O2-Dependent Hyperoxidation of Peroxiredoxin 6 (Prdx6) Plays a Role in Cellular Toxicity via Up-Regulation of iPLA2 Activity*. J. Biol. Chem..

[160] Rhee S.G., Chae H.Z., Kim K. (2005). Peroxiredoxins: A Historical Overview and Speculative Preview of Novel Mechanisms and Emerging Concepts in Cell Signaling. Free Radic. Biol. Med..

[161] Wood Z.A., Poole L.B., Karplus P.A. (2003). Peroxiredoxin Evolution and the Regulation of Hydrogen Peroxide Signaling. Science.

[162] Fisher A.B. (2018). The Phospholipase A2 Activity of Peroxiredoxin 6. J. Lipid Res..

[163] Manevich Y., Reddy K.S., Shuvaeva T., Feinstein S.I., Fisher A.B. (2007). Structure and Phospholipase Function of Peroxiredoxin 6: Identification of the Catalytic Triad and Its Role in Phospholipid Substrate Binding. J. Lipid Res..

[164] Manevich Y., Shuvaeva T., Dodia C., Kazi A., Feinstein S.I., Fisher A.B. (2009). Binding of Peroxiredoxin 6 to Substrate Determines Differential Phospholipid Hydroperoxide Peroxidase and Phospholipase A(2) Activities. Arch. Biochem. Biophys..

[165] Chen J.W., Dodia C., Feinstein S.I., Jain M.K., Fisher A.B. (2000). 1-Cys Peroxiredoxin, a Bifunctional Enzyme with Glutathione Peroxidase and Phospholipase A2 Activities. J. Biol. Chem..

[166] Li H., Benipal B., Zhou S., Dodia C., Chatterjee S., Tao J.-Q., Sorokina E.M., Raabe T., Feinstein S.I., Fisher A.B. (2015). Critical Role of Peroxiredoxin 6 in the Repair of Peroxidized Cell Membranes Following Oxidative Stress. Free Radic. Biol. Med..

[167] Fisher A.B. (2017). Peroxiredoxin 6 in the Repair of Peroxidized Cell Membranes and Cell Signaling. Arch. Biochem. Biophys..

[168] Jia W., Dong C., Li B. (2023). Anti-Oxidant and Pro-Oxidant Effects of Peroxiredoxin 6: A Potential Target in Respiratory Diseases. Cells.

[169] Wang H., Zhao Y., Zhou F., Chen F., Chen T., Wang J., Liu H., Sun C., Zhou R., Hu W. (2025). Peroxiredoxin 6: A Regulatory Target in Cellular Senescence and Age-Related Diseases. Antioxid. Redox Signal..

[170] zur Hausen H. (1991). Viruses in Human Cancers. Science.

[171] Dobo C., Oshima C.T.F., De Oliveira Lima F., Gomes T.S., Stávale J.N., Arias V., Ribeiro D.A., Focchi G.R.A. (2014). Cell-Cycle Analysis and Apoptosis-Associated Proteins in Cervical Lesions of Brazilian Women. Anticancer Res..

[172] Wheeler C.M. (2008). Natural History of Human Papillomavirus Infections, Cytologic and Histologic Abnormalities, and Cancer. Obstet. Gynecol. Clin. North Am..

[173] Wang W., Liu J., Feng Z., Hu W. (2026). From Genome Guardian to Immune Modulator: The Expanding Roles of Tumor Suppressor P53. Mol. Cell. Biol..

[174] Babamohamadi M., Babaei E., Ahmed Salih B., Babamohammadi M., Jalal Azeez H., Othman G. (2022). Recent Findings on the Role of Wild-Type and Mutant P53 in Cancer Development and Therapy. Front. Mol. Biosci..

[175] Reich N.C., Levine A.J. (1984). Growth Regulation of a Cellular Tumour Antigen, P53, in Nontransformed Cells. Nature.

[176] Haupt Y., Maya R., Kazaz A., Oren M. (1997). Mdm2 Promotes the Rapid Degradation of P53. Nature.

[177] Vousden K.H., Lane D.P. (2007). P53 in Health and Disease. Nat. Rev. Mol. Cell Biol..

[178] Clarke B., Chetty R. (2001). Cell Cycle Aberrations in the Pathogenesis of Squamous Cell Carcinoma of the Uterine Cervix. Gynecol. Oncol..

[179] Vazquez A., Bond E.E., Levine A.J., Bond G.L. (2008). The Genetics of the P53 Pathway, Apoptosis and Cancer Therapy. Nat. Rev. Drug Discov..

[180] Cheung T.H., Lo K.W., Yu M.M., Yim S.F., Poon C.S., Chung T.K., Wong Y.F. (2001). Aberrant Expression of P21(WAF1/CIP1) and P27(KIP1) in Cervical Carcinoma. Cancer Lett..

[181] Harper J.W., Elledge S.J., Keyomarsi K., Dynlacht B., Tsai L.H., Zhang P., Dobrowolski S., Bai C., Connell-Crowley L., Swindell E. (1995). Inhibition of Cyclin-Dependent Kinases by P21. Mol. Biol. Cell.

[182] Ter Harmsel B., Smedts F., Kuijpers J., Jeunink M., Trimbos B., Ramaekers F. (1996). BCL-2 Immunoreactivity Increases with Severity of CIN: A Study of Normal Cervical Epithelia, CIN, and Cervical Carcinoma. J. Pathol..

[183] Basu A., Haldar S. (1998). The Relationship between BcI2, Bax and P53: Consequences for Cell Cycle Progression and Cell Death. Mol. Hum. Reprod..

[184] Green D.R., Kroemer G. (2004). The Pathophysiology of Mitochondrial Cell Death. Science.

[185] Lopez J., Tait S.W.G. (2015). Mitochondrial Apoptosis: Killing Cancer Using the Enemy Within. Br. J. Cancer.

[186] Piaskowski S., Zawlik I., Szybka M., Kulczycka-Wojdala D., Stoczynska-Fidelus E., Bienkowski M., Robak T., Kusinska R., Jesionek-Kupnicka D., Kordek R. (2010). Detection of P53 Mutations in Different Cancer Types Is Improved by cDNA Sequencing. Oncol. Lett..

[187] Thomas M., Banks L. (1999). Human Papillomavirus (HPV) E6 Interactions with Bak Are Conserved amongst E6 Proteins from High and Low Risk HPV Types. J. Gen. Virol..

[188] Yang A., Kaghad M., Wang Y., Gillett E., Fleming M.D., Dötsch V., Andrews N.C., Caput D., McKeon F. (1998). P63, a P53 Homolog at 3q27-29, Encodes Multiple Products with Transactivating, Death-Inducing, and Dominant-Negative Activities. Mol. Cell.

[189] Kaghad M., Bonnet H., Yang A., Creancier L., Biscan J.C., Valent A., Minty A., Chalon P., Lelias J.M., Dumont X. (1997). Monoallelically Expressed Gene Related to P53 at 1p36, a Region Frequently Deleted in Neuroblastoma and Other Human Cancers. Cell.

[190] Yang A., Kaghad M., Caput D., McKeon F. (2002). On the Shoulders of Giants: P63, P73 and the Rise of P53. Trends Genet. TIG.

[191] Jost C.A., Marin M.C., Kaelin W.G. (1997). P73 Is a Simian [Correction of Human] P53-Related Protein That Can Induce Apoptosis. Nature.

[192] Lin Z., Liu M., Li Z., Kim C., Lee E., Kim I. (2006). DeltaNp63 Protein Expression in Uterine Cervical and Endometrial Cancers. J. Cancer Res. Clin. Oncol..

[193] Nekulova M., Holcakova J., Coates P., Vojtesek B. (2011). The Role of P63 in Cancer, Stem Cells and Cancer Stem Cells. Cell. Mol. Biol. Lett..

[194] Aubrey B.J., Kelly G.L., Janic A., Herold M.J., Strasser A. (2018). How Does P53 Induce Apoptosis and How Does This Relate to P53-Mediated Tumour Suppression?. Cell Death Differ..

[195] Lowe S.W., Cepero E., Evan G. (2004). Intrinsic Tumour Suppression. Nature.

[196] Hayflick L., Moorhead P.S. (1961). The Serial Cultivation of Human Diploid Cell Strains. Exp. Cell Res..

[197] Klepacki H., Kowalczuk K., Łepkowska N., Hermanowicz J.M. (2025). Molecular Regulation of SASP in Cellular Senescence: Therapeutic Implications and Translational Challenges. Cells.

[198] Feng W., Xiao J., Zhang Z., Rosen D.G., Brown R.E., Liu J., Duan X. (2007). Senescence and Apoptosis in Carcinogenesis of Cervical Squamous Carcinoma. Mod. Pathol..

[199] Khasawneh A.I., Al Shboul S., Himsawi N., Al Rousan A., Shahin N.A., El-Sadoni M., Alhesa A., Abu Ghalioun A., Khawaldeh S., Shawish B. (2025). Resolution of Oncogene-Induced Senescence Markers in HPV-Infected Cervical Cancer Tissue. BMC Cancer.

[200] Wright T.C., Ronnett B.M., Kurman R.J., Hedrick Ellenson L., Ronnett B.M. (2019). Benign Diseases of the Cervix. Blaustein’s Pathology of the Female Genital Tract.

[201] He Y., Qiu Y., Yang X., Lu G., Zhao S.-S. (2025). Remodeling of Tumor Microenvironment by Cellular Senescence and Immunosenescence in Cervical Cancer. Semin. Cancer Biol..

[202] Krizhanovsky V., Xue W., Zender L., Yon M., Hernando E., Lowe S.W. (2008). Implications of Cellular Senescence in Tissue Damage Response, Tumor Suppression, and Stem Cell Biology. Cold Spring Harb. Symp. Quant. Biol..

[203] Pérez-Mancera P.A., Young A.R.J., Narita M. (2014). Inside and out: The Activities of Senescence in Cancer. Nat. Rev. Cancer.

[204] Lasry A., Ben-Neriah Y. (2015). Senescence-Associated Inflammatory Responses: Aging and Cancer Perspectives. Trends Immunol..

[205] Shao H., Li X., Wu P., Chen Z., Zhang C., Gu H. (2023). A Cellular Senescence-Related Signature Predicts Cervical Cancer Patient Outcome and Immunotherapy Sensitivity. Reprod. Sci..

[206] Freund A., Patil C.K., Campisi J. (2011). p38MAPK Is a Novel DNA Damage Response-Independent Regulator of the Senescence-Associated Secretory Phenotype. EMBO J..

[207] Jones K.M., Bryan A., McCunn E., Lantz P.E., Blalock H., Ojeda I.C., Mehta K., Cosper P.F. (2024). The Causes and Consequences of DNA Damage and Chromosomal Instability Induced by Human Papillomavirus. Cancers.

[208] Coppé J.-P., Desprez P.-Y., Krtolica A., Campisi J. (2010). The Senescence-Associated Secretory Phenotype: The Dark Side of Tumor Suppression. Annu. Rev. Pathol. Mech. Dis..

[209] Prabhavathy D., Subramanian C.K., Karunagaran D. (2015). Re-Expression of HPV16 E2 in SiHa (Human Cervical Cancer) Cells Potentiates NF-κB Activation Induced by TNF-α Concurrently Increasing Senescence and Survival. Biosci. Rep..

[210] Faget D.V., Ren Q., Stewart S.A. (2019). Unmasking Senescence: Context-Dependent Effects of SASP in Cancer. Nat. Rev. Cancer.

[211] Ren C., Cheng X., Lu B., Yang G. (2013). Activation of Interleukin-6/Signal Transducer and Activator of Transcription 3 by Human Papillomavirus Early Proteins 6 Induces Fibroblast Senescence to Promote Cervical Tumourigenesis through Autocrine and Paracrine Pathways in Tumour Microenvironment. Eur. J. Cancer.

[212] Saleh T., Himsawi N., Al Rousan A., Alhesa A., El-Sadoni M., Khawaldeh S., Shahin N.A., Ghalioun A.A., Shawish B., Friehat K. (2024). Variable Expression of Oncogene-Induced Senescence/SASP Surrogates in HPV-Associated Precancerous Cervical Tissue. Curr. Issues Mol. Biol..

[213] Chen W., Huang S., Shi K., Yi L., Liu Y., Liu W. (2021). Prognostic Role of Matrix Metalloproteinases in Cervical Cancer: A Meta-Analysis. Cancer Control J. Moffitt Cancer Cent..

[214] Gonzalez-Meljem J.M., Apps J.R., Fraser H.C., Martinez-Barbera J.P. (2018). Paracrine Roles of Cellular Senescence in Promoting Tumourigenesis. Br. J. Cancer.

[215] Mukerjee N., Nag S., Bhattacharya B., Alexiou A., Mirgh D., Mukherjee D., Adhikari M.D., Anand K., Muthusamy R., Gorai S. (2024). Clinical Impact of Epithelial–Mesenchymal Transition for Cancer Therapy. Clin. Transl. Discov..

[216] Thomas M., Narayan N., Pim D., Tomaić V., Massimi P., Nagasaka K., Kranjec C., Gammoh N., Banks L. (2008). Human Papillomaviruses, Cervical Cancer and Cell Polarity. Oncogene.

[217] Ranieri D., French D., Raffa S., Guttieri L., Torrisi M.R., Belleudi F. (2021). Expression of the E5 Oncoprotein of HPV16 Impacts on the Molecular Profiles of EMT-Related and Differentiation Genes in Ectocervical Low-Grade Lesions. Int. J. Mol. Sci..

[218] Lu Y., Chen Y., Zhang Z., Li M., Chen X., Tu K., Li L. (2022). HPV16 E6 Promotes Cell Proliferation, Migration, and Invasion of Human Cervical Cancer Cells by Elevating Both EMT and Stemness Characteristics. Cell Biol. Int..

[219] Klymkowsky M.W., Savagner P. (2009). Epithelial-Mesenchymal Transition. Am. J. Pathol..

[220] Lee M.-Y., Chou C.-Y., Tang M.-J., Shen M.-R. (2008). Epithelial-Mesenchymal Transition in Cervical Cancer: Correlation with Tumor Progression, Epidermal Growth Factor Receptor Overexpression, and Snail up-Regulation. Clin. Cancer Res. Off. J. Am. Assoc. Cancer Res..

[221] Jiang J., Li X., Yin X., Zhang J., Shi B. (2019). Association of Low Expression of E-Cadherin and β-Catenin with the Progression of Early Stage Human Squamous Cervical Cancer. Oncol. Lett..

[222] Tian Y., Qi P., Niu Q., Hu X. (2020). Combined Snail and E-Cadherin Predicts Overall Survival of Cervical Carcinoma Patients: Comparison Among Various Epithelial-Mesenchymal Transition Proteins. Front. Mol. Biosci..

[223] Kumari K., Gupta R.K., Kumar S., Prasad S.B. (2025). Genes Associated with Epithelial Mesenchymal Transition (EMT) in Cervical Cancer Progression. Discov. Oncol..

[224] Gisca T., Matasariu D.R., Ursache A., Socolov D.G., Scripcariu I.-S., Fudulu A., Anton E.T.-T., Botezatu A. (2025). Integrating Biomarkers into Cervical Cancer Screening—Advances in Diagnosis and Risk Prediction: A Narrative Review. Diagnostics.

[225] Ozaki S., Zen Y., Inoue M. (2011). Biomarker Expression in Cervical Intraepithelial Neoplasia: Potential Progression Predictive Factors for Low-Grade Lesions. Hum. Pathol..

[226] Tornesello M.L., Buonaguro L., Giorgi-Rossi P., Buonaguro F.M. (2013). Viral and Cellular Biomarkers in the Diagnosis of Cervical Intraepithelial Neoplasia and Cancer. BioMed Res. Int..

[227] Onyango C.G., Ogonda L., Guyah B., Shiluli C., Ganda G., Orang’o O.E., Patel K. (2020). Novel Biomarkers with Promising Benefits for Diagnosis of Cervical Neoplasia: A Systematic Review. Infect. Agent. Cancer.

[228] Punhani P., Ahluwalia C. (2024). Biomarkers in the Screening and Management of Cervical Cancer. J. Colposc. Low. Genit. Tract Pathol..

[229] Zhang X., Shen D. (2018). p16INK4a and Ki-67 Measurement Predict Progression of Cervical Low-Grade Squamous Intraepithelial Lesion. Int. J. Clin. Exp. Pathol..

[230] Katerji M., Filippova M., Wongworawat Y.C., Siddighi S., Bashkirova S., Duerksen-Hughes P.J. (2020). Oxidative Stress Markers in Patient-Derived Non-Cancerous Cervical Tissues and Cells. Sci. Rep..

[231] Qadir A.M., Omar R.A. (2026). Oxidative Stress Biomarkers: Molecular Mechanisms, Detection Techniques, and Clinical Applications: A Comprehensive Review. J. Anal. Chem..

[232] Offor J.O., Okunade K.S., Iwalokun B.A., Oluwole A.A., Anorlu R.I. (2021). Evaluation of Oxidative Markers in Women with Invasive Cervical Cancer in Lagos, Nigeria. Ecancermedicalscience.

[233] Shah S., Kalal B.S. (2019). Oxidative Stress in Cervical Cancer and Its Response to Chemoradiation. Turk. J. Obstet. Gynecol..

[234] Siegel E.M., Patel N., Lu B., Lee J.-H., Nyitray A.G., Craft N.E., Frenkel K., Villa L.L., Franco E.L., Giuliano A.R. (2012). Biomarkers of Oxidant Load and Type-Specific Clearance of Prevalent Oncogenic Human Papillomavirus Infection: Markers of Immune Response?. Int. J. Cancer.

[235] Shrivastava A., Mishra S.P., Pradhan S., Choudhary S., Singla S., Zahra K., Aggarwal L.M. (2021). An Assessment of Serum Oxidative Stress and Antioxidant Parameters in Patients Undergoing Treatment for Cervical Cancer. Free Radic. Biol. Med..

[236] Jelic M., Mandic A., Maricic S., Bozin B., Kladar N., Sudji J., Conic B.S. (2023). Predictive Power of Oxidative Stress Biomarkers in Recurrence and Survival in Advanced Cervical Cancer. Exp. Oncol..

[237] Salzman R., Pácal L., Tomandl J., Kanková K., Tóthová E., Gál B., Kostrica R., Salzman P. (2009). Elevated Malondialdehyde Correlates with the Extent of Primary Tumor and Predicts Poor Prognosis of Oropharyngeal Cancer. Anticancer Res..

[238] Zahra K., Patel S., Dey T., Pandey U., Mishra S.P. (2021). A Study of Oxidative Stress in Cervical Cancer- an Institutional Study. Biochem. Biophys. Rep..

[239] Romano G., Sgambato A., Mancini R., Capelli G., Giovagnoli M.R., Flamini G., Boninsegna A., Vecchione A., Cittadini A. (2000). 8-Hydroxy-2’-Deoxyguanosine in Cervical Cells: Correlation with Grade of Dysplasia and Human Papillomavirus Infection. Carcinogenesis.

[240] Jelić M., Mandić A., Kladar N., Sudji J., Božin B., Srdjenović B. (2018). Lipid Peroxidation, Antioxidative Defense and Level of 8-Hydroxy-2-Deoxyguanosine in Cervical Cancer Patients. J. Med. Biochem..

[241] Yamazaki H., Inoue T., Koizumi M., Tanaka E., Yoshioka Y., Nakamura H., Shuo X., Inoue T. (2005). Urinary 8-Hydroxy-2’-Deoxyguanosine Excretion as a Biomarker for Estimating DNA Oxidation in Patients Undergoing External Radiotherapy and/or Brachytherapy. Oncol. Rep..

[242] Lin H.-Y., Zhu X., Mazumder H.O.R., Ronis M., Pedersen K.B., Hagensee M. (2024). Serum Oxidative Biomarkers Associated with Genital HPV Infection and Cervical Lesions in Women. J. Med. Virol..

[243] Arip M., Tan L.F., Jayaraj R., Abdullah M., Rajagopal M., Selvaraja M. (2022). Exploration of Biomarkers for the Diagnosis, Treatment and Prognosis of Cervical Cancer: A Review. Discov. Oncol..

[244] Chambers C.R., Ritchie S., Pereira B.A., Timpson P. (2021). Overcoming the Senescence-Associated Secretory Phenotype (SASP): A Complex Mechanism of Resistance in the Treatment of Cancer. Mol. Oncol..

[245] Tjiong M.Y., van der Vange N., ten Kate F.J.W., Tjong-A-Hung S.P., ter Schegget J., Burger M.P.M., Out T.A. (1999). Increased IL-6 and IL-8 Levels in Cervicovaginal Secretions of Patients with Cervical Cancer. Gynecol. Oncol..

[246] Vahedpour Z., Abedzadeh-Kalahroudi M., Sehat M., Piroozmand A., Memar M. (2021). Comparison of Cervical Levels of Interleukins-6 and -8 in Patients with and without Cervical Intraepithelial Neoplasia. Asian Pac. J. Cancer Prev. APJCP.

[247] Chen X., Lin L., Wu Q., Li S., Wang H., Sun Y. (2023). Tumor Necrosis Factor-α Promotes the Tumorigenesis, Lymphangiogenesis, and Lymphatic Metastasis in Cervical Cancer via Activating VEGFC-Mediated AKT and ERK Pathways. Mediat. Inflamm..

[248] Dobbs S.P., Hewett P.W., Johnson I.R., Carmichael J., Murray J.C. (1997). Angiogenesis Is Associated with Vascular Endothelial Growth Factor Expression in Cervical Intraepithelial Neoplasia. Br. J. Cancer.

[249] Ghosh A., Moirangthem A., Dalui R., Ghosh T., Bandyopadhyay A., Dasgupta A., Banerjee U., Jana N., Basu A. (2014). Expression of Matrix Metalloproteinase-2 and 9 in Cervical Intraepithelial Neoplasia and Cervical Carcinoma among Different Age Groups of Premenopausal and Postmenopausal Women. J. Cancer Res. Clin. Oncol..

[250] Xu H., Niu M., Yuan X., Wu K., Liu A. (2020). CD44 as a Tumor Biomarker and Therapeutic Target. Exp. Hematol. Oncol..

[251] Svanadze T., Turashvili T., Kepuladze S., Burkadze G. (2025). P16 and CD44 as Biomarkers for Predicting the Progression of Immature Polypoid Squamous Metaplasia of the Cervix. Cureus.

[252] Mehdi H.K., Raju K., Sheela S.R. (2023). Association of P16, Ki-67, and CD44 Expression in High-Grade Squamous Intraepithelial Neoplasia and Squamous Cell Carcinoma of the Cervix. J. Cancer Res. Ther..

